# Chemically stabilized long antisense oligonucleotides perturb RNA exosome function

**DOI:** 10.1093/nar/gkag990

**Published:** 2026-10-15

**Authors:** Daniel Tobias Hofacker, Stefanie Gackstatter, Vanessa Deisling, Thorsten Stafforst

**Affiliations:** Interfaculty Institute of Biochemistry, University of Tübingen, 72076Tübingen,Germany; Interfaculty Institute of Biochemistry, University of Tübingen, 72076Tübingen,Germany; Interfaculty Institute of Biochemistry, University of Tübingen, 72076Tübingen,Germany; Interfaculty Institute of Biochemistry, University of Tübingen, 72076Tübingen,Germany; Cluster of Excellence iFIT (EXC 2180) ‘Image-Guided and Functionally Instructed Tumor Therapies’, University of Tübingen, 72076Tübingen,Germany; Gene and RNA Therapy Center (GRTC), Faculty of Medicine University, 72076Tübingen,Germany

## Abstract

The interaction with intracellular proteins shapes the pharmacology and toxicity of RNA drugs like gapmers and siRNAs. Here, we identify the RNA exosome as a modification-sensitive interaction partner of long, chemically stabilized, single-stranded RNA drugs. Using proximity biotinylation proteomics, we observe preferential binding of the catalytic exosome subunits DIS3 and EXOSC10 to oligonucleotides stabilized by 2′-ribose modifications and phosphorothioate linkages. RNA drug binding correlates with exosome impairment as measured by dose-dependent stabilization of promoter upstream transcripts and accumulation of ribosomal RNA processing intermediates leading to reduced cell viability. Consistent with structural requirements for substrate threading through the exosome channel, oligonucleotides longer than 24 nucleotides can impair exosome function. Systematic variation of ribose and backbone chemistry reveals that exosome inhibition is highly sensitive to the spatial arrangement of chemical modifications and can be mitigated by incorporation of bulky 2′-MOE residues or alternative internucleoside linkages like mesyl phosphoramidate. In contrast to the clinically established RNA drug modalities, class-specific liabilities of emerging ADAR-recruiting oligonucleotides remain poorly understood. Importantly, we demonstrate that ADAR-recruiting oligonucleotides can be designed that preserve editing activity while minimizing exosome interference. These findings illuminate critical variables that must be considered in the safer design of next-generation RNA base editing therapeutics.

## Graphical Abstract

**Graphical Abstract:**
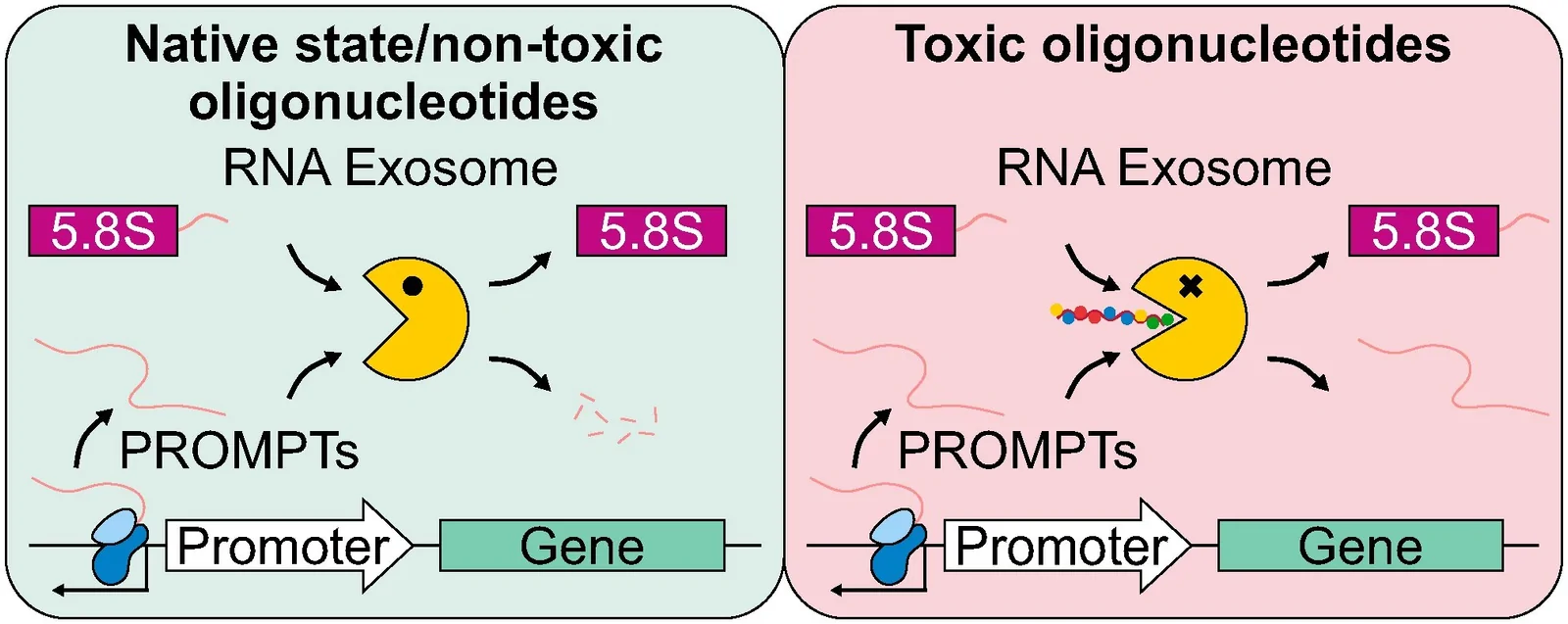

## Introduction

Therapeutic antisense oligonucleotides (ASOs) display a vastly emerging field with >20 RNA drugs approved by the FDA today [1]. Lately, site-directed RNA base editing gained attention as a novel class of RNA therapeutics. This ASO class harnesses the endogenous adenosine deaminases acting on RNA (ADAR1 and ADAR2) [2, 3] to rewrite genetic information at the RNA level by site-directed adenosine-to-inosine (A-to-I) deamination. Already a few years after the first description of RNA base editing platforms, the first-ever clinical data in a therapeutic setting demonstrated efficacy and tolerability in human [4]. For efficient recruitment of ADAR, the oligonucleotides typically have a length of ≥30 nt and are asymmetrically placed on the target adenosine and require a complex, varied chemical modification pattern at the ribose moieties and internucleoside linkages [3, 5, 6]. This distinguishes ADAR-recruiting oligonucleotides from other therapeutic RNA classes like gapmer ASOs, siRNAs, and splice-switching oligonucleotides (SSOs), which are typically shorter in length (gapmer ASOs: 14–20 nt, siRNAs: typically 21/23 nt per strand, SSOs: 18–30 nt) and contain simpler chemical modification patterns [1], while using the same modifications in principle. These include 2′-fluoro (2′-F), 2′-O-methoxyethyl (2′-MOE), 2′-O-methyl (2′-OMe), sometimes 2′-4′ locked nucleic acids (LNA), and the internucleoside linkage chemistries phosphodiester (PO), phosphorothioate (PS,*), mesyl phosphoramidate (&), and phosphoryl guanidine (PN,#).

An ASO’s modification pattern shapes the pharmacological properties of the RNA drug. First of all, modifications are introduced to stabilize the RNA drug metabolically in order to increase effect duration. To avoid loss in efficacy, they must be introduced carefully, which requires the development of RNA drug class-specific modification patterns (e.g. specific patterns for gapmer ASOs, siRNAs, and ADAR-recruiting oligonucleotides). Modifications furthermore modulate protein binding, which influences biodistribution, delivery, cellular uptake, potency, and efficiency, but also adverse effects like systemic and cellular toxicity and immunogenicity (reviewed in Crooke *et al*. and Shen *et al*. [7, 8]). While the mode-of-action differs tremendously for the different classes, RNA drugs share general toxicity pathways. In general, RNA drug toxicity can occur sequence- and hybridization-dependent, independent of both, or may be dependent on the sequence but not on hybridization [9]. Thus, sequence and modification pattern both contribute to RNA drug toxicity [10–12]. Especially in the field of gapmer ASOs, several pathways leading to cellular toxicity have been described in the last decade. For example, gapmers with 2′-fluoro-modified wings were found to cause degradation of paraspeckle proteins—namely SFPQ and NONO—upon their direct binding [13]. This correlated well with hepatotoxicity [14]. Moreover, ASOs carrying bridged nucleic acids are commonly known for their tendency to accumulate inside the nucleoli [15]. Nucleolar localization of toxic ASOs was later connected to protein mis-localization of SFPQ and NONO mediated through enhanced interactions with RNase H1 [16]. These were based on conformational changes of RNase H1 [17], and nucleolar accumulation of the SFPQ/NONO/RNase H1 complex was ultimately connected to perturbed ribosomal RNA processing [7, 12, 16]. Here, the therapeutic index could be improved by replacing specific chemical modifications, either at the ribose [12] or by replacing the PS linkage with alkyl phosphonate [11], highlighting the importance of the chemical modification pattern to reduce ASO toxicity. For the clinically established classes of RNA drugs (e.g. gapmers, siRNAs, and SSOs), specific, tolerable modification patterns have been identified and can often be transferred to new targets given their specific mode of action. For the class of ADAR-recruiting oligonucleotides, this knowledge is yet to be generated. Given the different length and modification requirements, class-specific effects are well conceivable for this novel class of RNA drugs.

In this study, we report a modification-dependent interaction between ADAR-recruiting oligonucleotides and the RNA exosome complex. The exosome is an essential protein complex that is responsible for the targeted degradation of RNA [18]. Two 3′→5′ exonuclease subunits, EXOSC10 and DIS3, are oppositely located at the top and bottom of the nine-subunit barrel structure of the exosome. In most pathways [19], the RNA substrate is fed through the exosome barrel into the catalytic site of DIS3, where the RNA is degraded from its 3′-end into mono nucleotides [20, 21]. On the other hand, EXOSC10 seems to play a major role in the processing and biogenesis of ribosomal RNA and thus is mainly localized to the nucleolus. The exosome is responsible for the degradation of defective mRNA—that are, for example, identified by ribosome stalling [22]—but also “unwanted” transcripts like promoter upstream transcripts (PROMPTs) [23]. The latter are quickly degraded by-products of transcription since many RNA polymerase II promoters are bidirectional and contain upstream antisense initiation sites of transcription within a 500–2500-nt window upstream the transcription start site [24–26]. Their transcription regularly fails transition to the elongation phase and thus PROMPTs are typically rather short (200–600 nt) [23, 27]. PROMPTs are directed to the exosome through the NEXT complex [28] and are stabilized upon knockdown or depletion of exosome components like DIS3 or structural components like EXOSC3 or EXOSC5 [21, 23].

Here, we show that RNA drugs can bind to the RNA exosome, in particular the exosome components EXOSC10 and DIS3, and inhibit their function in a dose-dependent manner. This effect occurs for oligonucleotides longer than ~24 nt, depends strongly on the modification pattern, and may at least partially explain observed RNA drug toxicity. Mechanistically, we speculate that the nuclease-resistant RNA drug is inserted into and clogs the exosome’s central channel thereby inhibiting its function.

## Materials and methods

### Synthesis of BG-oligonculeotides, Atto488-oligonucleotides, and FISH probes

Oligonucleotides were obtained from Biospring or AIRNA Inc. in HPLC-purified quality. All oligonucleotide sequences and their modification patterns can be found in Supplementary Data file S1. The PolyU-FISH probe was obtained from Sigma–Aldrich in desalted quality carrying two amino linkers (5′-C6-amino linker and 3′-C7-amino linker). For linker attachment, the oligos were ordered with a 5′-C6-amino linker. The BG moiety was attached and the oligonucleotides purified by polyacrylamide gel electrophoresis (PAGE) as described previously in detail [29]. Fluorescently labeled oligonucleotides and the FISH probe were prepared and purified by precipitation as described recently [30]. The labeling efficiency was calculated by measuring the absorbance at 260 and 500 nm (ε_500nm_ = 90 mM^−1^cm^−1^) and was higher than 90% for all oligonucleotides.

### Cell culture

Cells were cultivated in Dulbecco’s modified Eagle’s medium (DMEM; Thermo Fisher Scientific) with 10% fetal bovine serum (FBS; Thermo Fisher Scientific) in a humidified atmosphere at 37°C and 5% CO_2_. For sub-cultivation and seeding, cells were washed with phosphate buffered saline (PBS) and detached with 0.25% trypsin/EDTA (Sigma–Aldrich) and resuspended in fresh DMEM/FBS. Cells were split every 2–4 days.

For SILAC experiments, cells were labeled with lysine- and arginine-deficient DMEM (PAN-Biotech) supplemented with 10% dialyzed FBS (PAN-Biotech) and 1% penicillin–streptomycin as described recently [30]. The “light” medium additionally contained ^12^C_6_, ^14^N_2_  l-lysine (Sigma–Aldrich) and ^12^C_6_, ^14^N_4_  l-arginine (Sigma–Aldrich). D_4_  l-lysine (Cambridge Isotope Laboratories) and ^13^C_6_  l-arginine (Silantes) were added to the “medium” condition and ^13^C_6_, ^15^N_2_  l-lysine (Cambridge Isotope Laboratories) and ^13^C_6_, ^15^N_4_  l-arginine (Silantes) were added to the “heavy” medium. Cells were cultivated for at least 10 days before the experiment. For splitting and seeding, SILAC cells were washed twice with PBS and detached with 0.5 mM ethylenediaminetetraacetic acid (EDTA; Sigma–Aldrich) until being fully detached.

### Transfection

All transfections were performed using Lipofectamine RNAimax (Thermo Fisher Scientific) following the manufacturer’s instructions. Briefly, oligonucleotides or siRNAs were diluted in Opti-MEM (10% of the final volume in the well) and RNAimax (1.5 µl/24-well) was diluted in Opti-MEM (10% of the final volume in the well) and incubated for 5 min. Then, the oligonucleotide mixture and RNAimax dilutions were mixed at equal volumes and incubated for 15 min before applying the mixtures to the cells. For all other well formats, the volumes were adjusted accordingly.

### Immunofluorescence

For immunofluorescence experiments, 50 000 Hela wild-type cells were seeded onto 12-mm coverslips in a 24-well plate. Twenty-four hours later, the indicated oligonucleotide concentration was transfected as described above. For each transfection, 2 nM Atto-488 labeled oligonucleotide was spiked-in to compare co-localization of the protein and the oligonucleotide. Another 24 h later, cells were fixed with 3.7% formaldehyde for 10 min at room temperature, washed with PBS, permeabilized with 0.1% Triton X-100/PBS for 10 min at room temperature, washed again with PBS, and blocked for 1.5 h with 1% bovine serum albumin (BSA) in PBS at 4°C. The primary antibodies were diluted 1:200 in 0.5% BSA/PBS and incubated overnight. The next day, the samples were washed once with PBS and the secondary antibody was applied (1:1000 in PBS) together with NucBlue staining (Thermo Fisher Scientific, 1:100) for 1 h at room temperature. Cells were washed three times and then mounted with fluorescent mounting medium. All used antibodies can be found in Supplementary Table S1.

### Fluorescence microscopy

All microscopic images were acquired on a Nikon Eclipse Ti-2 inverted fluorescent microscope with a photometrics Prime 95B camera and a Lumencor AURA II light engine. For all experiments, a 60× oil objective (MRD31605, NA 1.4) with Olympus IMMOIL-F30CC immersion oil was used. To record the individual channels, the following excitation and filter settings were used: Atto488: 475 nM excitation light, excitation filter: Chroma ET500/×20, beamsplitter: Chroma Chroma T515lp, emission filter: Chroma ET535/30m. Atto594: 575 nM excitation light, excitation filter: Semrock 585/29 BrightLine HC, beamsplitter: Chroma T610LPXR, emission filter: Semrock 650/60 BrightLine HC. DAPI: 390 nM excitation light, excitation filter: Chroma 89402x, beamsplitter: Chroma 89402bs, emission filter: Chroma 89402m. For all images, z-stacking covering 6 µm (0.2 µm steps) was applied and the images deconvoluted using the Nikon NIS Offline deconvolution 4.51 software. A maximum projection over three layers was performed with ImageJ. In each sub-figure, the same acquisition and contrast settings were applied and linear lookup tables used for each channel.

### isASO-ID

isASO-ID was performed as published recently [30] with minor changes. Briefly, four wells on a six-well plate with 250 000 cells per well were seeded per condition for one replicate. Twenty-four hours post-seeding, oligonucleotides were transfected at the indicated concentrations using 7.5 µl Lipofectamine RNAimax/well using SILAC medium without FBS. Another 24 h later, cells were fixed with 3.7% formaldehyde (10 min, room temperature), washed with PBS, and permeabilized with 0.1% Triton X-100 (10 min, room temperature). After washing twice with PBS, SNAP-BASU-His (240 nM) was conjugated for 1 h at 37°C in 1 ml conjugation buffer (2.5% glycerol, 50 mM NaCl, 10 mM Tris–HCl, 1 mM dithiothreitol) per well. Unconjugated enzyme was washed away with PBS and the biotinylation reaction buffer was added for 24 h (2 mM β-mercaptoethanol, 1.5 mM ATP, 1.5 mM MgCl_2_, 50 µM biotin in 10 mM sodium bicarbonate in PBS, pH 8.3). The reaction was stopped by two washes with PBS and cells were subsequently harvested in 225 µl colorless Laemmli buffer per well using a cell scraper. Crosslinking was reversed by heating the samples to 98°C for 1 h. Prior to the streptavidin pulldown, samples were diluted three-fold in PBS supplemented with one tablet protease inhibitor cocktail (Roche) per 25 ml. An overview of all isASO-ID experiments in this study can be found in Supplementary Table S2.

### Biotin pulldown

Enrichment of biotinylated proteins was performed as published recently [30]. The samples with different labels (L, M, H) were pooled in equal amounts and incubated overnight at 4°C on an end-over-end rotator (15 rpm) with 200 µl Streptavidin Magnetic Sepharose (GE Healthcare) per experiment. After removing the supernatant, beads were washed twice with wash buffer 1 [2% sodium dodecyl sulfate (SDS), all washing steps with 500 µl, 5 min, at room temperature], one with wash buffer 2 [0.1% (w/v) deoxycholate, 1% (w/v) Tween 20, 350 mM NaCl, 1 mM EDTA, pH 8.0], once with wash buffer 3 [0.5% (w/v) deoxycholate, 0.5% (w/v) Tween 20, 1 mM EDTA, 250 mM LiCl, 10 mM Tris–HCl, pH 7.4], once with wash buffer 4 (50 mM NaCl, 50 mM Tris–HCl, pH 7.4), and three times with 50 mM ammonium bicarbonate. Biotinylated proteins were eluted by addition of 20 µl elution buffer (10 mM Tris, pH 7.4, 2% SDS, 5% β-mercaptoethanol, and 2 mM biotin) and boiling at 98°C for 15 min. This procedure was repeated and the supernatants were combined [31]. Then 6× Lämmli buffer was added and the samples were boiled for 5 min to 95°C. The samples were subsequently loaded onto an 8%–16% Tris–glycine Mini Gel (Thermo Fisher Scientific). The gels were run until the bromophenol band migrated ~2.5 cm into the gel. Proteins were fixed using ReadyBlue Protein Gel Stain (Sigma–Aldrich) for 2 h and destained with water. Lanes were excised with a scalpel and the intense streptavidin band (running at ~17 kDa) was separated and processed independently.

### In-gel digest and mass spectrometry

Coomassie-stained gel pieces were digested using trypsin [32] and desalted [33]. Subsequently, the samples were analyzed on an Easy-nLC 1200 coupled to a Q Exactive HF mass spectrometer (Thermo Fisher Scientific) as described previously [30, 34] with slight modifications. Peptide mixtures were separated using a 57-min gradient of 10%-33%-50%-90% of HPLC solvent B (80% acetonitrile in 0.1% formic acid) in HPLC solvent A (0.1% formic acid) at a flow rate of 200 nl/min. At each scan cycle, the seven (Q Exactive HF) most intense precursor ions were sequentially fragmented using higher-energy collisional dissociation fragmentation. The sequenced precursor ions were then excluded from further selection for 30 s. As target values for MS/MS fragmentation, 10^5^ charges and for the MS scan, 3 × 10^6^ charges were chosen.

MaxQuant software package version 1.6.14.0 was used to process the acquired MS spectra with the integrated Andromeda search engine [35, 36]. A target-decoy database from *Homo sapiens* was obtained from uniprot (downloaded on 7 October 2020) and used for database search. Additionally, data were searched against SNAP-BASU-His. For database search, full trypsin digestion specificity was required and up to two missed cleavages were allowed. As modification, carbamidomethylation was set as fixed and N-terminal acetylation and oxidation of methionine were set as variable modifications. The initial precursor mass tolerance was set to 4.5 ppm and 20 ppm at the MS/MS level was. Lys4/Arg6 and Lys8/Arg10 were defined as medium and heavy labels, respectively. A minimum of two quantified peptides was required for protein group quantitation. The false discovery rate for peptide, protein, and modification site identification was estimated by the target-decoy approach [37] and reported at 0.01. Scatter plots were created with Perseus software (version 1.6.15.0) and modified with CorelDraw 2017. All SILAC experiments are listed in Supplementary Table S2.

### Western blot

For western blotting, cells were lysed in 1× RIPA buffer supplemented with one tablet protease inhibitor cocktail (Roche) per 10 ml and 6× Laemmli buffer was added. The samples were boiled for 15 min at 98°C and loaded onto an 8%–16% Tris–glycine Mini Gel (Thermo Fisher Scientific). The gel was run until the bromophenol band reached the lower edge of the gel. Protein samples were transferred onto a PVDF membrane using a wet transfer system. The membrane was activated in methanol and equilibrated in transfer buffer (25 mM Tris hydrochloride, 190 mM glycine, 20% methanol). Proteins were transferred for 16 h at 30 V. The membrane was washed once with TBST and blocked with 5% BSA (Sigma–Aldrich) in TBST for 1 h at room temperature. The primary and secondary antibodies were diluted in 2.5%–5% non-fat dry milk in TBST as listed in Supplementary Table S1. After antibody incubation, the membrane was washed three times with TBST. Signals were developed using Western Blot ECL solution (1.25 mM Luminol, 0.2 mM p-coumaric acid, 100 mM Tris–HCl, pH 8.5) with freshly added 20% H_2_O_2_ (1:1000) and chemiluminescence was detected using a Licor Odyssey Fc imaging system (Licor). For additional information on antibodies, see Supplementary Table S1.

### RNA isolation, DNase digest, bioanalyzer, reverse transcription, and qPCR

For all qPCR experiments, 75 000 Hela cells were seeded per well in a 24-well plate and transfected the next day as described above. Twenty-four hours later prior to RNA isolation, cells were lysed in RLT buffer (Qiagen) and RNA was purified using the Monarch RNA Cleanup kit (New England Biolabs) following the manufacturer’s instructions. Three micrograms of RNA was used for DNase digest using the TURBO DNase (Thermo Fisher Scientific) according to the manufacturer’s protocol. RNA integrity was measured using the Agilent bioanalyzer 2100 and the RNA nano kit following the manufacturer’s instructions after DNase digest. RNA integrity numbers were obtained directly from the software. For reverse transcription, 10 µl of the supernatant after TURBO DNase treatment was used using the High Capacity Reverse Transcription kit (Thermo Fisher Scientific). The transcribed complementary DNA (cDNA) was purified using the NucleoSpin® Gel and PCR Clean-up kit (Macherey-Nagel) and diluted to 10 ng/µl. qPCR was performed with the Fast SYBR Green master mix (Thermo Fisher Scientific) and 10 ng cDNA template was added per well in a 384-well plate. All samples were measured in two to three technical replicates. *ACTB* and *GAPDH* served as internal reference genes. All primer sequences are listed in Supplementary Table S3. Additional information on primer pair efficiencies is available in the Appendix of the Supplementary Information. The qPCR was run on a QuantStudio 7 system (Thermo Fisher Scientific). The program consisted of an initial denaturation step (95°C, 20 s), followed by 40 cycles of denaturation (95°C, 3 s), and annealing/elongation (60°C, 30 s). A baseline correction was performed for each dataset using the QuantStudio Real-Time PCR Software (Thermo Fisher Scientific) before determining the C(t) values. PROMPT expression for each sample was referenced to the geometric mean of *ACTB* and *GAPDH* using Microsoft Excel 2021. To calculate ΔC(t), the geometric mean C(t) obtained with *ACTB* and *GAPDH* primers was subtracted from the average C(t) obtained with the primers specific for each PROMPT. ΔΔC(t) values and fold change (2−ΔΔC(t)) were determined in comparison to untreated cells from the ΔC(t) obtained for each treatment. Results were displayed with GraphPad Prism 8.

### Cell viability assay

To determine the cell viability, the CellTiter-Glo® 2.0 Cell Viability Assay (Promega) was used to measure intracellular ATP. Five thousand Hela cells were seeded in a black well 96-well plate in DMEM/FBS. After 24 h, cells were transfected as described above (0.4 µl Lipofectamine RNAimax/well) at the indicated concentrations. Cell viability was measured at the indicated time points. Therefore, before the measurement, the plate and the CellTiter-Glo reagent were allowed to reach room temperature and 100 µl of the CellTiter-Glo reagent was added per well. The plate was shaken for 2 min (600 rpm) on an orbital shaker and incubated for another 10 min. Luminescence was recorded with a Spark 10 M plate reader (Tecan) and the data normalized to untreated cells after background subtraction using Excel 2021. All experiments were performed in triplicates. The data were plotted using GraphPad Prism 8.

### PolyA FISH

For PolyA-FISH experiments, cells were seeded on microscopy slides in a 24-well plate as described above. Cells were fixed with 3.7% formaldehyde (10 min, room temperature) 24 h after oligoucleotide transfection and subsequently permeabilized with 0.1% Triton X-100 (10 min, room temperature) and 100% ethanol (>4 h, 4°C. Samples were re-hydrated in FISH wash buffer (10% formamide, 2× SSC) for 10 min at room temperature and the fluorescent PolyU probe was hybridized overnight at 37°C in hybridization buffer (15 nM in 10% formamide, 10% dextran sulfate, 2× SSC). The next day, the samples were washed twice with FISH wash buffer (10 min, 37°C) and stained with DAPI (1:100 in PBS, 1 h, room temperature) before mounting and microscopy.

### RNA editing analysis

Sixty-five thousand HeLa PiggyBac SERPINA1 cells were seeded in 500 µl in a 24-well plate. Cells were transfected 24 h after seeding with 50 nM oligonucleotide and harvested 24 h post-transfection. Cells were lysed and RNA isolated as described for qPCR. Remaining DNA was removed by DNase I treatment (New England Biolabs). For each sample, 2 µg RNA was digested with 1 µl DNase I for 30 min at 37°C following the manufacturer’s protocol. The reaction was stopped by adding 3 µl Na-EDTA (25 mM) and heat inactivation for 10 min at 75°C. For target amplification, the OneTaq One-Step RT-PCR kit (New England Biolabs) was used following the company’s protocol. First, the reverse primer, sense stand, 0.25 µl dimethyl sulfoxide (DMSO), and 3 µl of nuclease free water were combined with 5 µl RNA and denatured for 1 min at 95°C, before adding the forward primer, One-step reaction mix, and the enzyme mix. Reverse transcriptase-polymerase chain reaction (RT-PCR) was performed according to the manufacturer’s instructions. Annealing was performed at 54°C and elongation time was set to 30 s. The PCR product was purified by gel electrophoresis. Samples were mixed with 6× DNA loading dye and loaded onto a 1.4% agarose gel with RotiStain (Carl ROTH) for DNA visualization and separated for 30 min in TAE buffer (120 V). Bands were excised and purified with the NucleoSpin® Gel and PCR Clean-up kit (Macherey-Nagel).

Twelve microliters of sample (15 ng/µl) and 3 µl of the reverse primer (10 µM) were sent for sequencing (Microsynth). The editing efficiency was calculated by dividing the difference of the edited peak height with the sum of the edited peak and the unedited peak height. All primer sequences are listed in Supplementary Table S4.

### Statistical analysis

Statistical analysis was performed using GraphPad Prism 8.4.3. Before statistical analysis, all data normalized to a reference were transformed by log_2_. To determine statistical significance in the cell viability assay and the concentration row of PROMPT expression (Figs 1A and 2C), a two-way mixed-effects model with oligonucleotide and concentration as fixed factors and experiment as matched factor was used (Geisser-Greenhouse correction for sphericity). Pairwise comparisons between oligonucleotides at individual concentrations were performed using Tukey’s multiple-comparisons test. For all comparisons with the reference oligonucleotide #317 or the untreated condition, a two-tailed one sample *t*-test was performed. In each comparison group, values were corrected with the Holm–Šídák method. The results are displayed in Figs 2A and 3A. For all other comparisons, a one-way ANOVA with repeated measures and each row as matched set was performed using a mixed-effects model. Sphericity was assumed and pairs for comparison were selected and Šídák’s correction was performed. The results are displayed in Figs 4, 5, and 6.

## Results

### The wing chemistry of ADAR-recruiting oligonucleotides determines RNA exosome binding and correlates with reduced cell viability

Protein binding of RNase H1-recruiting gapmers has been studied extensively (for review see Crooke *et al*. [7]) and specific protein–RNA interactions could be connected to class-specific toxicities. We were wondering how far the same or other interactions are relevant for the emerging class of ADAR-recruiting oligonucleotides. Although this novel class of RNA drugs makes use of the same modifications than established RNA therapeutics, the modification patterns massively differ from those commonly used in gapmers, siRNAs, or SSOs. We and others recently published design strategies to optimize on-target effects using only [6] or mainly [38] commercially available modifications. It turned out that a mixed, pretty variable 2′-modification pattern (e.g. 2′-F, 2′-H, 2′-OMe, and 2′-MOE) was beneficial to improve editing yields, although several hotspot sites could be identified that prefer very specific modifications. In this study, we furthermore identified the protein interactome of two ADAR-recruiting oligonucleotides and found many RNA and DNA binding proteins formerly shown to bind to RNA drugs of other classes. However, when we recently re-visited our former lead design (#414, which corresponds to oligonucleotide #317 from Pfeiffer *et al*. [6] with an additional 5′ amino linker for the isASO-ID assay [30]) for antitrypsin deficiency and estimated its cellular toxicity in an ATP-dependent cell viability assay, we observed a clear dose-dependent reduction of cell viability 48 h after transfection (Fig. 1A). Specifically, cell viability was reduced by 43% when transfecting 50 nM oligo #414. Notably, this effect was much weaker within the first 24 h after transfection (Supplementary Fig. S1) indicting a slow mechanism of cellular toxicity. The observed loss in cell viability was between that of an approved RNA drug (Mipomersen) and that of a toxic gapmer control [12]. Notably, the observed toxic effect could be clearly traced back to the 3′-wing modification (three consecutive 2′-O-methyl) in oligo #414 since another oligo, #494, also carrying three consecutive 2′-O-methyl modifications at the 3′- but not at the 5′-terminus showed a similar reduction in cell viability as well. In contrast, cell viability was much less affected (minus 25% at 50 nM oligo transfection) when the otherwise identical oligonucleotide contained three consecutive 2′-MOE modifications at the 3′-end (oligo #467) or both termini (oligo #466).

**Figure 1.**
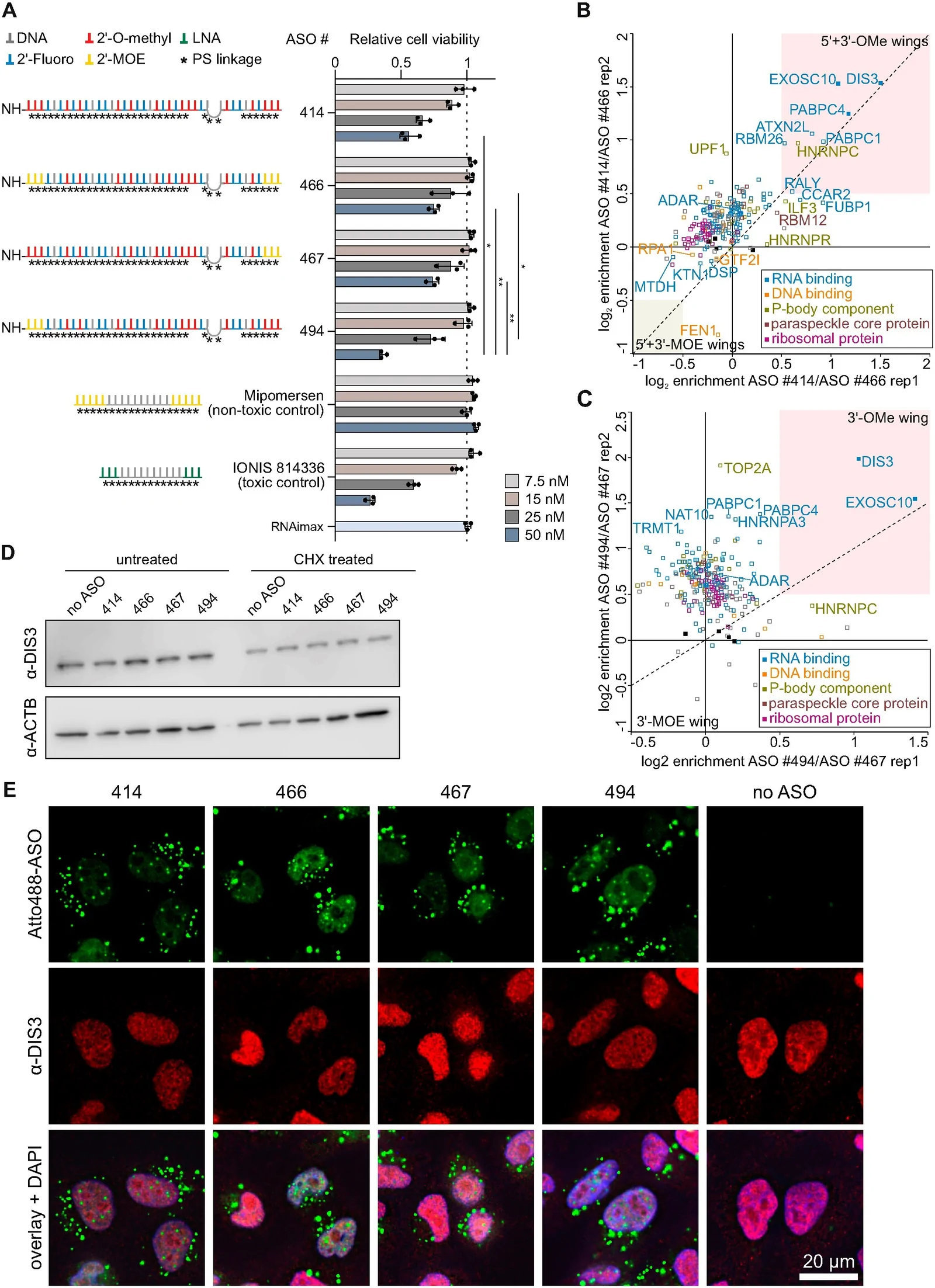
The toxicity of ADAR-recruiting oligonucleotides correlated with exosome complex binding with respect to their 2′-modifications. (**A**) Cell viability assay 48 h after oligo transfection. Shown are mean values and individual data points and the standard deviation. All signals were normalized to untreated cells. As controls, two gapmer ASOs (non-toxic and toxic) were included. *N* = 3. Asterisks mark significance (two-way mixed-effects model with Geisser-Greenhouse correction) as indicated by lines. *P < .05, **P < .01. (**B**) SILAC-MS/MS for 25 nM oligo #414 against 25 nM oligo #466. 15 nM oligo were BG-modified and 10 nM oligo were transfected as unmodified NH-oligo. Plotted was the enrichment (log_2_) of replicate 1 against replicate 2 with swapped SILAC labeling. The red box indicates an enrichment >0.5 log_2_ units in both replicates for oligos carrying a 3′-OMe wing. The green box highlights an enrichment >0.5 log_2_ units in both replicates for oligos carrying a 3′-MOE wing. Proteins were colored according to their GO terms. (**C**) SILAC-MS/MS for 25 nM oligo #494 against 25 nM oligo #467. Plotted was the enrichment (log_2_) of replicate 1 against replicate 2 with swapped SILAC labeling. (**D**) Western blot against DIS3 24 h after transfection with 50 nM of the indicated oligo. Additionally, cells were treated with CHX to inhibit global translation. As loading control, a western blot against ACTB was performed. (**E**) Immunofluorescence microscopy against DIS3 24 h after transfection with 50 nM of the indicated oligonucleotide with 2 nM Atto488-labeled oligo spiked-in to investigate co-localization of DIS3 and the oligonucleotide.

We were wondering how far these differences could be traced back to the protein interactome of the respective oligonucleotides. Thus, we applied our recently developed *in situ* ASO-ID proximity biotinylation assay [30] to the oligonucleotides containing either three O-methyl (oligo #414) or three MOE (oligo #466) modifications at both wings (Fig. 1B and Supplementary Fig. S2A and B). The protein interactomes of both oligonucleotides was very similar and revealed numerous known and well-described (gapmer) ASO-binding proteins, including members of the HNRNP family, the DBHS proteins NONO, and SFPQ, but also ADAR itself (Supplementary Fig. S2A and B). Surprisingly, when plotting oligo #414 (both wings O-methyl-modified) against #466 (both wings MOE-modified), the majority of proteins was biotinylated to a similar level, but a few proteins that preferentially bound to the 2′-O-methyl-modified oligo caught our attention (Fig. 1B). Among these, the two proteins showing the highest enrichment, DIS3 and EXOSC10, display a 3′→5′-exoribonuclease activity. Thus, we speculated that solely the 3′-terminus of oligo #414 was responsible for the enhanced binding of the two exonucleases, and we conducted another MS experiment with two oligonucleotides (#467 and #494) containing now one O-methyl and one MOE wing in swapped orientation, respectively (Fig. 1C and Supplementary Fig. S2C and D). Again—and this time even exclusively—the two proteins that revealed an enrichment >0.5 log_2_ units toward the 2′-OMe-modified 3′-terminus were DIS3 and EXOSC10 showing that indeed the chemical modification at the 3′-wing contributed strongly to their binding. Notably, similar as oligo #414 and #466, oligo #467, with the 3′-MOE-modified wing, was less toxic in the cell viability assay than oligo #494, containing the 3′-O-methyl-modified wing (Fig. 1A).

### Oligonucleotide binding does not interfere with stability or subcellular localization of DIS3 and EXOSC10

It was reported in the literature that certain toxic gapmer ASOs (e.g. when rich in 2′-F/PS) degrade some proteins they interact with (like NONO [13, 14]). Thus, we wanted to see whether the interaction of our oligonucleotides (e.g. #414) has any influence on the stability of DIS3 or EXOSC10. For this, we performed a western blot against DIS3 and EXOSC10 after transfection of four different oligonucleotides at 50 nM, however, we did not observe a clear difference for any of the four oligonucleotides tested compared to an empty transfection control, neither on the protein levels of DIS3 (Fig. 1D) nor EXOSC10 (Supplementary Fig. S3A). This result also did not change when we inhibited global translation by adding 50 µg/ml cycloheximide (CHX) (Fig. 1D) [13]. On other cases, gapmer ASO-mediated toxicity did not rely on protein degradation, but subcellular protein mislocalization to the nucleoli [16]. We performed fluorescence immunostaining of DIS3 (Fig. 1E) and EXOSC10 (Supplementary Fig. S3B) after transfection with 50 nM partially Atto488-labled oligonucleotides to investigate whether the interaction with the oligonucleotide affects intracellular protein localization. In all cases, no obvious shift was visible and DIS3 remained distributed in the whole nucleus, while EXOSC10 was localized predominantly in the nucleoli irrespective of oligo transfection. Thus, neither degradation nor mislocalization of the exosome or its components was observed and could explain the reduced cell viability of oligonucleotides carrying a 3′-OMe-modified terminus.

### Oligonucleotide binding leads to dysregulation of ribosomal RNA processing

After ruling out degradation or mislocalization of the exosome or its components, the binding of the oligonucleotides to DIS3 and EXOSC10 might interfere with exosome function, like RNA degradation and processing. During rRNA biogenesis, EXOSC10 and DIS3 act alone or in combination with the RNA exosome complex to catalyze different maturation steps [39]. It was reported previously that RNA drugs can influence ribosomal RNA (rRNA) processing and expression [12, 40]. But to our knowledge, only mislocalization of DBHS proteins in complex with RNase H1 in a gapmer ASO-dependent manner has been described in detail today [16, 17]. In other studies, defects in rRNA processing were connected to nucleolar SFPQ localization and direct binding of SFPQ to rRNA upon cellular stress [41]. However, DIS3 and in particular EXOSC10 play crucial roles in rRNA maturation (for review, see Pirouz *et al*. [39]), for example, they remove the 3′-terminal internal transcribed spacer 2 (ITS2) from the 12S pre-rRNA and contribute to its processing to the 5.8S rRNA and degrade the ITS1 fragment after cleavage from the 45S pre-rRNA. Thus, we investigated the expression of two regions within mature rRNA (5.8S and 18S) and the two ITS regions (ITS1 and ITS2), which are only present in maturation intermediates of rRNAs by RT-qPCR, thus probing rRNA processing and expression (Fig. 2A). Specifically, we compared three different oligos; one SSO (#615) that was reported to perturb rRNA processing and increase pre-rRNA expression [40], our lead oligo against antitrypsin deficiency (#317), and a reported toxic gapmer ASO (IONIS 814336). An ASO similar to the latter was described to alter rRNA levels through its interaction with DBHS proteins and RNase H1 [12]. For our lead design (#317), we observed unchanged expression levels in both regions from mature rRNA (5.8S and 18S) 24 h after transfection, but an increase in ITS fragment abundance. The ITS2 sequence, which is part of the 12S precursor of the 5.8S rRNA—and which is trimmed by EXOSC10 and DIS3—was increased more than two-fold (Fig. 2A, *P* = .0317). Further, we observed a significant upregulation of the ITS1 fragment present inter alia in the 45S pre-rRNA, by 1.7-fold (*P* = .0022). The data for the selected SSO #615 (a 25nt PS/2′-OMe oligonucleotide) looked very similar to oligo #317, with a moderate upregulation of ITS fragments, well in accordance with the original literature report [40]. A similar observation was made by others when knockdown of single exosomal proteins led to an upregulation of pre-rRNA species [42]. In contrast only a weak upregulation of ITS species (<1.4-fold) was observed for the toxic gapmer ASO (IONIS 814336). This is again in accordance with literature [12] where the ASO gapmer rather induced a downregulation of the 47S pre-rRNA species. This indicates that the different classes of RNA drugs can interfere differently with rRNA processing. However, at the indicated oligo doses the RNA quality, as determined based on rRNA occurrence via a bioanalyzer experiment (Fig. 2B and Supplementary Fig. S4) did not suffer (RNA integrity number was >9.5). This is in contrast to the literature report [42] on SSO #615 and may be due to the lower oligonucleotide dose, different cell types, and high stability of rRNAs.

**Figure 2.**
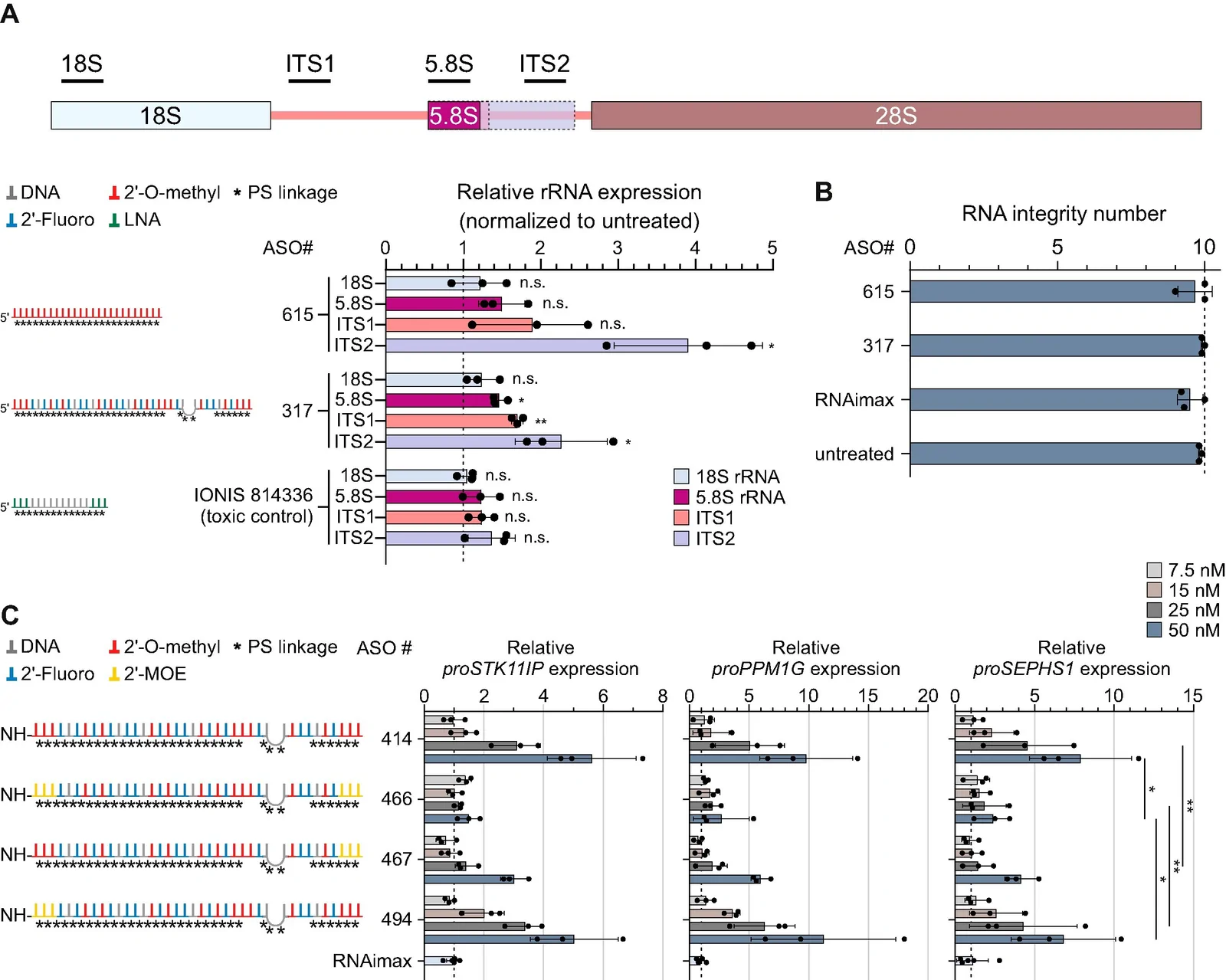
Oligonucleotide transfection influences rRNA expression and leads to an upregulation of PROMPTs. (**A**) Relative rRNA expression after transfection with 50 nM of the respective oligo for 24 h. Placement of the primer pairs is displayed schematically above. Relative rRNA expression levels were referenced to *ACTB* and *GAPDH* expression in each sample and the relative expression was calculated with the ΔΔCt method and normalized to untreated cells. Asterisks mark significance (two-tailed one sample *t*-test) against the untreated control. n.s. = not significant, *P < .05, **P < .01. *N* = 3. (**B**) RNA integrity after 50 nM oligo transfection for 24 h. The raw data can be found in Supplementary Fig. S4. *N* = 3. (**C**) Relative PROMPT expression after transfection of ADAR-recruiting oligonucleotides. The indicated oligos were transfected at increasing concentrations and RNA levels were analyzed 24 h later via qPCR. Relative PROMPT expression was referenced to *ACTB* and *GAPDH* expression in each sample and the relative expression was calculated with the ΔΔCt method and normalized to untreated cells. Shown is the mean with individual data points and the standard deviation. *N* = 3–5. Asterisks mark significance of the geometric mean over all three analyzed PROMPTs per replicate (two-way mixed-effects model with Geisser-Greenhouse correction) as indicated by lines. *P < .05, **P < .01.

### Oligonucleotide transfection leads to dose-dependent PROMPT accumulation

Given the rather weak effect of our oligonucleotides on rRNA processing, we next focused on their effect on the degradation of transcription by-products upstream of RNA polymerase II promoters. PROMPT degradation is typically carried out by the whole RNA exosome complex—including both exoribonucleases DIS3 and EXOSC10—and depletion of exosome components was shown to lead to PROMPT stabilization [21, 23]. We picked three well-known PROMPTs (*proSTK11IP, proPPM1G*, and *proSEPHS1*) [23] and determined their expression levels in absence and presence of increasing amounts of four oligonucleotides by RT-qPCR. Treatment with oligo #414 carrying 2′-OMe wings increased PROMPT expression in a dose-dependent manner up to 10-fold (Fig. 2C). The same picture was observed for oligo #494 (with 3′-terminal 2′-OMe wing), whereas the oligo #467 and oligo #466 (2′-MOE wing 3′- or 3-′ and 5′-terminal) caused only a minor PROMPT stabilization (Fig. 2C). Notably, this trend perfectly reflects the interaction of the given oligos with DIS3/EXOSC10 observed using isASO-ID (Fig. 1B and C) and furthermore correlates very well with the respective reduction of cell viability (Fig. 1A). The data suggest that the strong interaction of some oligonucleotides with the exosome at least partly inhibits exosome function and contributes to their cellular toxicity profile.

### PROMPT stabilization depends on oligonucleotide length

RNA substrates can be processed by the exoribonucleases alone or in concert with the full exosome complex. The substrate requirements are well studied. DIS3, for example, binds and processes oligonucleotides with a single-stranded overhang >10 nt and directly binds to up to 16 nt of its RNA substrate [43–45]. The minimal RNA substrate length for exosomal degradation, however, is around 24 nt [44, 46] and a total length of around 30–32 nt is spanned by DIS3 and the central channel [47]. The rules for substrate specificity should enable to discriminate whether a chemically modified oligonucleotide inhibits either the nuclease directly or needs to occupy the entire exosomal substrate binding site. To test for this, we shortened our lead oligo #317 stepwise from the 5′-end from 40 nt down to 10 nt and analyzed PROMPT expression levels after transfection (Fig. 3A). Notably, oligonucleotides shorter than 24 nt did not elicit any significant PROMPT accumulation. Between a length of 24 nt and 30 nt, PROMPT expression levels steeply increased to achieve the maximal PROMPT expression already at 30 nt, which was the same as found for the 40 nt oligo #317. These data support a mechanism where the oligonucleotide clogs the exosome substrate channel and disfavors a direct inhibitory mechanism of the isolated nucleases.

**Figure 3.**
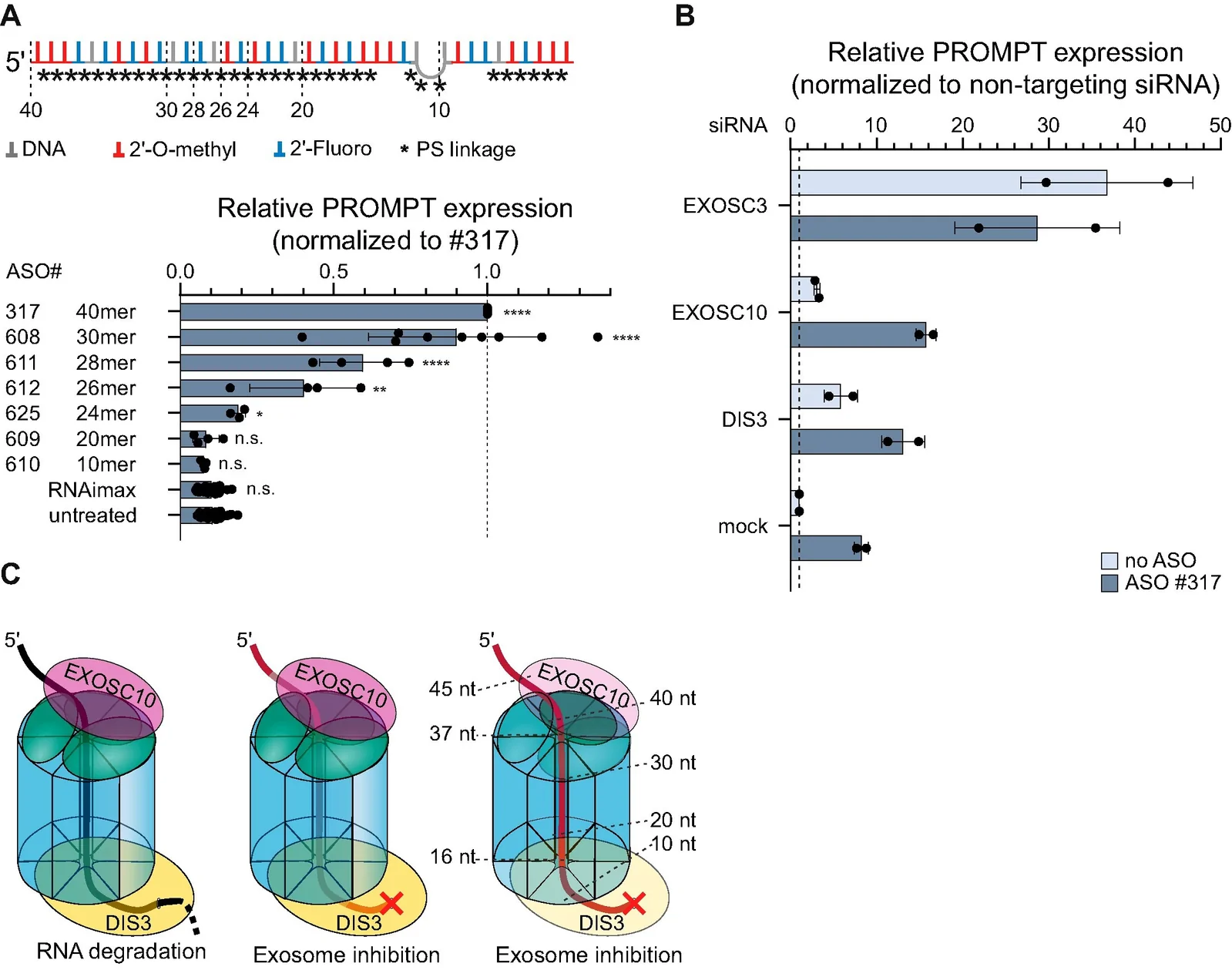
Oligonucleotides bind and impair the full exosome complex resulting in PROMPT accumulation. (**A**) Relative PROMPT expression for different oligos (50 nM transfection) shortened from the 5′-end using the ΔΔCt method, normalized to the 40-mer reference oligo #317. The geometric mean of all three PROMPT expression levels was calculated. Oligos were stepwise shortened from the 5′-end, while the 3′-end was kept unchanged in sequence and modification pattern. Asterisks mark significance (two-tailed one sample *t*-test) against the untreated control. n.s. = not significant, *P < .05, **P < .01, ***P < .001, ^****^P < .0001. (**B**) Relative PROMPT expression levels after knockdown of the RNA exosome components EXOSC3, EXOSC10, and DIS3, respectively with siRNAs for 6 days. On day 5, oligo #317 was transfected additionally at a final concentration of 50 nM where indicated. The geometric mean of all three PROMPT expression levels was calculated. (**C**) Proposed mechanism of RNA exosome inhibition by long, chemically stabilized oligonucleotides. Left panel: the natural RNA substrate (black) is guided through the central channel of the RNA exosome and degraded by the exoribonuclease activity of DIS3. Middle and right panel: The oligonucleotide (red) replaces the natural substrate and thus blocks and inhibits the RNA exosome. The positioning of an RNA substrate, or oligonucleotide, respectively, in the full exosome complex is indicated, calculated from the well-defined 3′-terminal binding of the RNA substrate to the catalytic site in the DIS3 subunit [43].

To further test this hypothesis, we established the siRNA knockdown of the nucleases DIS3 and EXOSC10 but also a structural component of the exosome, EXOSC3 (efficiency and specificity were tested by RT-qPCR and western blot, see Supplementary Fig. S5). Knockdown of the structural component EXOSC3 had the strongest effect on PROMPT abundance (Fig. 3B) and the additional transfection of oligo #317 added no further effect on PROMPT levels. Knockdown of EXOSC10 and DIS3 had much weaker effects on PROMPT stabilization. Interestingly, the effect of EXOSC10 was the least on PROMPT levels but allowed a strong induction of PROMPT accumulation by additional transfection of oligo #317. Together, these data reinforce our hypothesis that the chemically modified oligonucleotide stably binds the entire exosome complex and inhibits its function due to its engineered nuclease resistance (Fig. 3C). However, to verify this hypothesis, an *in vitro* RNA degradation assay using a reconstituted exosome complex would be required.

Recently, it was further reported that knockdown of the RNA exosome complex leads to perturbation of global RNA homeostasis and massive mRNA upregulation [48]. This effect was traced back to the entire complex rather than the ribonucleases themselves, and knockdown of the structural compounds EXOSC3 and EXOSC5 caused more severe upregulation of polyadenylated RNA compared to EXOSC10 and DIS3, respectively. We observed a similar effect after oligonucleotide transfection (Supplementary Fig. S6). Oligonucleotide treatment led to a massive increase in PolyA FISH signal. Notably, this was only the case for oligo #317 and #608 (40 and 30 nt) that also elicited PROMPT levels, but this was not the case for the shortened oligo #609 (20 nt) that did not elicit PROMPT levels. Surprisingly, after transfection of oligo #317 or #608, FISH signal was enhanced more prominently in the cytosol in contrast to the FISH signal upon knockdown of EXOSC3 where the signal was predominantly visible in the nucleus. The cytosolic mRNA speckles induced by oligonucleotide transfection might be stress granules.

### Interference with RNA exosome activity is modulated by chemical modification pattern of the oligonucleotide

Long (>24 nt) chemically stabilized oligonucleotides may bind to and seem to inhibit the exosome contributing to their toxicity profile. Our data showed that chemical modifications modulate this. For example, bulky modifications like 2′-MOE at the 3′-terminus can reduce interference with exosome activity and improve cell viability. To better understand which regions in an oligonucleotide and which modification patterns affect exosome interference, we screened numerous oligos with regard to their effect on PROMPT stabilization (Figs 4 and 5, and Supplementary Fig. S7). More specifically, we re-used various, recently published ADAR-recruiting RESTORE-ASOs [6], which vary in their length (30–59 nt), ribose, and linkage modification pattern.

**Figure 4.**
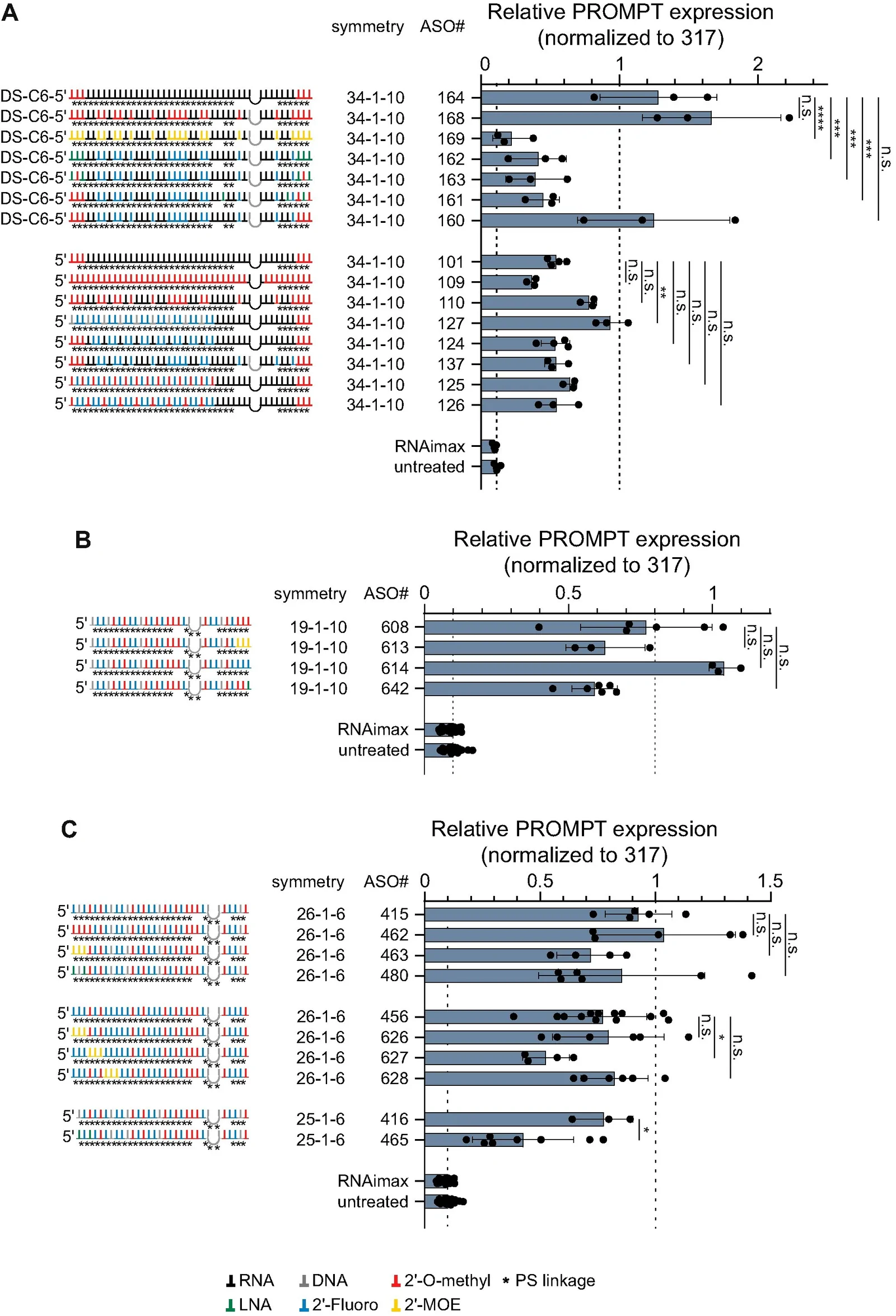
RNA exosome inhibition is sensitive to 2′-ribose modifications at several positions in the oligonucleotide. (**A**) Relative PROMPT expression levels for 45-mer *GAPDH*-targeting oligonucleotides. Internal expression normalization was performed against *ACTB* and then expression levels were normalized to oligonucleotide #317 PROMPT expression levels. (**B**) Relative PROMPT expression levels for oligonucleotides containing different 3′-wing modifications. (**C**) Relative PROMPT expression levels for oligonucleotides containing different 5′-wing modifications. In all panels, all oligonucleotides were transfected to a final concentration of 50 nM. Shown is the mean with individual data points and the standard deviation. For all oligos, the symmetry is displayed as 5′-binding region-counter base-binding region-3′. Asterisks mark significance (one-way ANOVA with a mixed effects model) as indicated by lines. n.s. = not significant, *P < .05, **P < .01, ***P < .001, ^****^  *p* < 0.0001.

**Figure 5.**
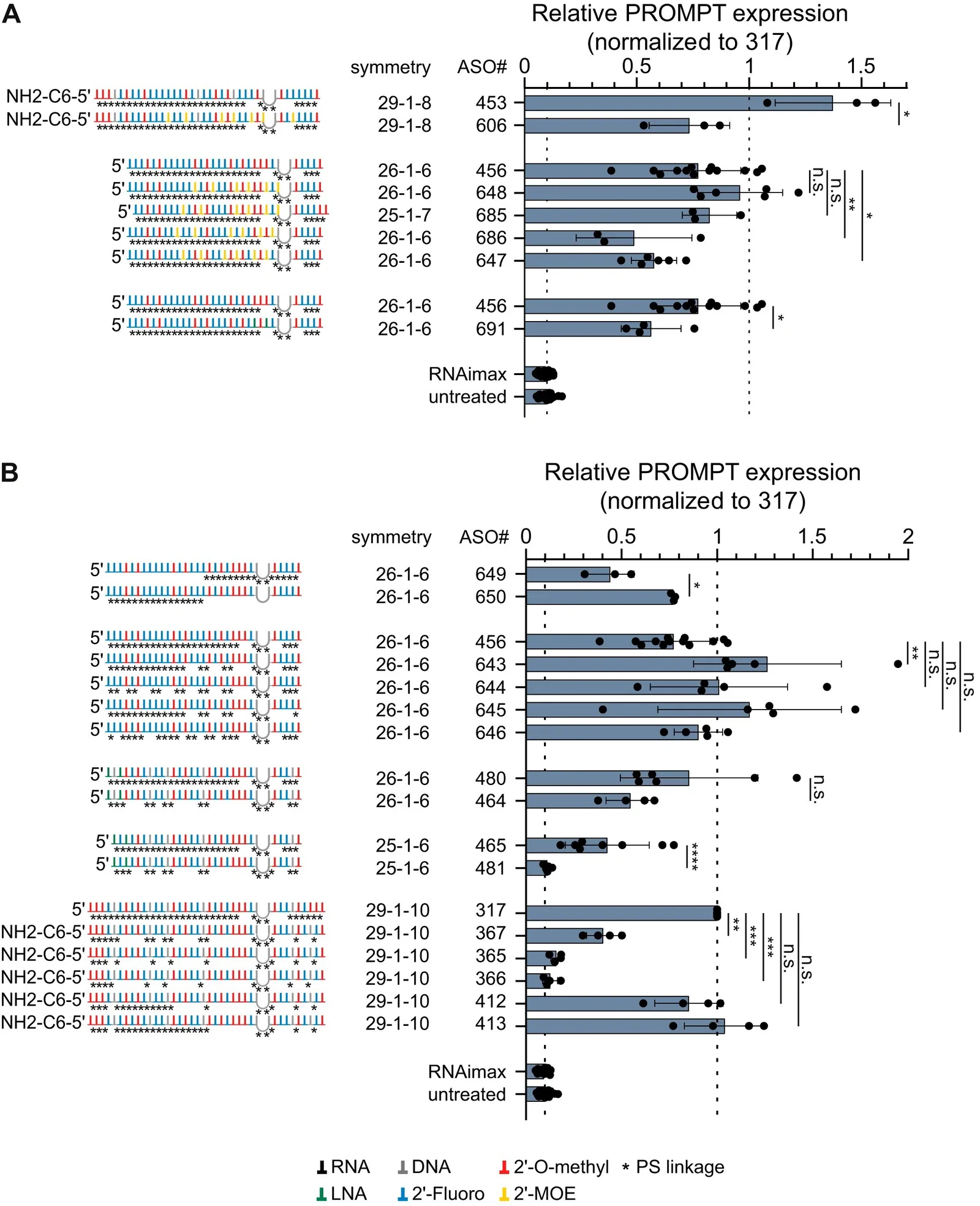
RNA exosome inhibition is mediated through internal PS and 2′MOE modifications. (**A**) Relative PROMPT expression levels for oligonucleotides containing different internal modifications, mainly 2′-MOE but also LNA. (**B**) Relative PROMPT expression levels for oligonucleotides with different PS content/distribution. In all panels, all oligonucleotides were transfected to a final concentration of 50 nM. Shown is the mean with individual data points and the standard deviation. For all oligos, the symmetry is displayed as 5′-binding region-counter base-binding region-3′. Asterisks mark significance (one-way ANOVA with a mixed effects model) as indicated by lines. n.s. = not significant, *P < .05, **P < .01, ***P < .001, ^****^p < .0001. For comparisons with #317, a two-tailed *t*-test was performed.

In a first step, we compared the effect of common 2′-modifications, which are used in approved RNA drugs, or which are used in clinical trials, including 2′-OMe, MOE, LNA, DNA, and F. All oligonucleotides were 45 nt long and heavily modified with PS linkages to obtain stability against nucleases. In this context, 2′-OMe (oligo #168) turned out to strongly inhibit the RNA exosome (Fig. 4A). This is not surprising since 2′-OMe was recognized to inhibit the DIS3-related bacterial 3′-5′ exoribonuclease R from *Mycoplasma genitalium* [49, 50]. Furthermore, EXOSC10 was shown to be modification-sensitive and to be inhibited by a 3′-terminal phosphate [51]. However, replacing 2′-hydroxy by 2′-OMe or by introducing 2′-F did not enhance exosome inhibition overall compared to 2′-OMe end blocks alone (#168 and 160 versus 164). In contrast, after introduction of bulky 2′-MOE at the same positions (#169), PROMPT levels were significantly reduced (*P* < .0001) and almost at the level of untreated cells. A similar, slightly weaker effect was observed when sterically demanding LNA was added in the termini (#161–163, *P* < .001 for all oligos compared to #164). When the placement of 2′-OMe, 2′-F, and RNA was varied, typically no big differences in PROMPT levels were determined. A fully 2′-OMe modified oligonucleotide inhibited the RNA exosome slightly less and 2′-F and DNA showed the strongest inhibitory effect (Fig. 4A). We found the same trends (2′-F/OMe strong versus 2′-MOE/LNA weak inhibitory) in a 30 nt oligonucleotide, however, the effect size was smaller (Fig. 4B). Introduction of single MOE modifications either close to the 3′- or 5′-terminus in 30 nt and 33 nt long oligonucleotides, in contrast, did not lead to any notable reduction in exosome inhibition (Supplementary Fig. S7A). Hence, not a single hotspot site is responsible for the beneficial effect of 2′-MOE but rather the synergistic effect of multiple modifications.

Since the interactions between an RNA substrate and the RNA exosome cover up to 45 nucleotides (Supplementary Fig. S8, [43, 45]), we further focused on the 5′-wing and internal modifications. For the 5′-wing, similar trends were observed as for the 3′-wing before. Again, 2′-MOE led to a slight reduction, but only when placed at nucleotide positions 28–30 (counting from the 3′-end, oligo #627, *P* = .0233), whereas the other positions tested (31–33 and 25–27, oligo #626 and #628) had no influence (Fig. 4C). Surprisingly, site-specific introduction of LNA modifications at nucleotide positions 30 and 32 (oligo #465) kept exosome inhibition weak (*P* = .0045), whereas LNA at nucleotide positions 31 and 33 (oligo #480) did not interfere with PROMPT upregulation (Fig. 4B/C). Thus, this effect seems to be position-specific as well. Interestingly, when placing six or seven internal 2′-MOE modifications their effect on reducing PROMPT expression compared to their parental designs was either good [Fig. 5A, e.g. #453 and 606 (*P* = .0004) as well as #456 and 686 (*P* = .0019)] or bad (#456 versus 648 and 685) even though the 2′-MOE pattern was only slightly shifted. Furthermore, we investigated the effect of the total DNA level on PROMPT expression. For the *SERPINA1* targeting oligonucleotides, we saw a trend that DNA reduction correlates with lower PROMPT levels in most cases (#371 and 388), whereas for CRB1 no such trend was observed (Supplementary Fig. S7B). Overall, it seems as if the site of a specific modification and not only its pure content plays a crucial role in modulating exosome inhibition. Interestingly, oligonucleotides targeting different mRNAs that otherwise shared the same symmetry and modification pattern, showed up to six-fold differences in PROMPT expression (Supplementary Fig. S7C and S7D, #426, 399, and 443 as well as #291, 319, 320, and 327). This effect could not be traced back to their overall base content but rather the sequence (Supplementary Fig. S7D). Scrambling the sequence and modification pattern of our standard oligonucleotide #317 (Supplementary Fig. S7E) led to a similar outcome and a reduction of PROMPT levels to 66% of the reference design (*P* = .0006).

Structural analysis shows strong interactions between the RNA exosome and its bound RNA substrate via the phosphodiester backbone (Supplementary Fig. S8). It is well known from the literature that PS linkages heavily influence and typically stabilize protein binding [52–54]. Accordingly, we tested the effect of various PS modification patterns on PROMPT stabilization. We first compared two 33-nt long oligonucleotides that either contained 16 consecutive PS linkages in their 5′-half (oligo #650) or in their 3′-half (#649), respectively. Surprisingly, oligo #650 carrying PS in its 5′-half showed much stronger inhibition than oligo #649 (*P* = .0447, Fig. 5B). It seems that a high PS content within the region of the oligonucleotide that is presumably bound to the exosome channel contributes more to exosome inhibition than the PS linkages in the region that binds to the DIS3 subunit. The exosome channel may provide more space and more conformational flexibility to the RNA substrate to enable this. Next, we tested several different PS patterns where we removed increasing numbers of PS linkages that presumably interact with the RNA exosome, but no strong effects were observed (Fig. 5B, #456, 643–646). Nevertheless, the overall reduction of the PS content led to a decrease in PROMPT stabilization (#317, 367, 365, and 366). Again, a high PS content within the 5′-half was sufficient for inhibition (#412 and 413). Notably, the beneficial effect from PS reduction (in the 5′-half) was synergistic to the one observed when placing LNA at the 5′-wing (#465 and 481, *P* < .0001, Fig. 5B).

Finally, we studied the exosome inhibition of the recently published AImer platform [3], however, in a stereo-random version of the oligonucleotides. These oligonucleotides contain a fully 2′-F-modified 5′-side and a fully 2′-OMe-modified 3′-side. Overall, compared to the RESTORE oligonucleotides the AImer-like designs equally induced PROMPT accumulation (Supplementary Fig. S7C), indicating that these long, chemically modified oligonucleotides in general tend to bind and inhibit the exosome.

### Efficient ADAR-recruiting oligonucleotides can be designed that do not impair exosome function

From our current understanding, effective ADAR-recruiting RNA drugs need to have >24 nt and thus are capable of binding and inhibiting the exosome. We were wondering whether it was possible to generate oligonucleotides that are chemically fully modified, but still efficient in recruiting ADAR and that furthermore had low tendency to induce PROMPT accumulation (Fig. 6). Specifically, we tested several oligonucleotides that contain complex 2′-modification patterns including 2′-F, 2′-OMe, and with a high content of bulky 2′-MOE as well as different backbone modifications including PO and PS but also sterically demanding linkage modifications like mesyl phosphoramidate or phosphoryl guanidine. These molecules (#564, 566, 568, 570) were highly efficient in ADAR recruitment and concurrently showed a very mild RNA exosome inhibition not exceeding 25% of our reference oligo #317 (Fig. 6A). Their good performance was partially traced back to the overall 2′-modification pattern (#683) and partially to the 2′-MOE pattern (#684) as well as a to the overall low PS content. Incorporation of six mesyl phosphoramidate (#564) or six phosphoryl guanidine linkages (#566) further lowered PROMPT levels close to the level of untreated cells. Both linkage modifications are bulkier and more hydrophobic than PS and thus may reduce binding affinity or even block the loading of the RNA drug into the central channel of the RNA exosome. However, whilst the editing efficiency remained high (~75%) when replacing several PS modifications by mesyl phosphoramidate, it dropped significantly to around 50% when PS was either replaced by PO or PN (Fig. 6B). When we kept the six mesyl phosphoramidates but removed all seven 2′-MOE modifications (#788) the PROMPT accumulation was only slightly stronger compared to #564 but clearly lower than #683 (no 2′-MOE, no mesyl phosphoramidates, Fig. 6A). The effect of mesyl phosphoramidate and 2′-MOE seemed to be cumulative (#564 versus #788/#568 and #683) but less position-specific since removing a few mesyl phosphoramidate and 2′-MOE modifications elevated PROMPT levels similarly, regardless of whether they were removed at one of the termini or in the center of the oligonucleotide (#789–791). In this case, editing levels were also preserved at ∼75% in all cases (Fig. 6B). Notably, #564 also did not alter cellular mRNA levels as verified by FISH (Supplementary Fig. S5). The standard PS design (#568) was subsequently transferred to two other sequences (*STAT1* and *CRB1*, #690 and 689, respectively) inducing a similarly low level of PROMPTs (Fig. 6A). Overall, efficient, chemically stabilized ADAR-recruiting oligonucleotides can be designed that do not interfere with exosome activity.

**Figure 6.**
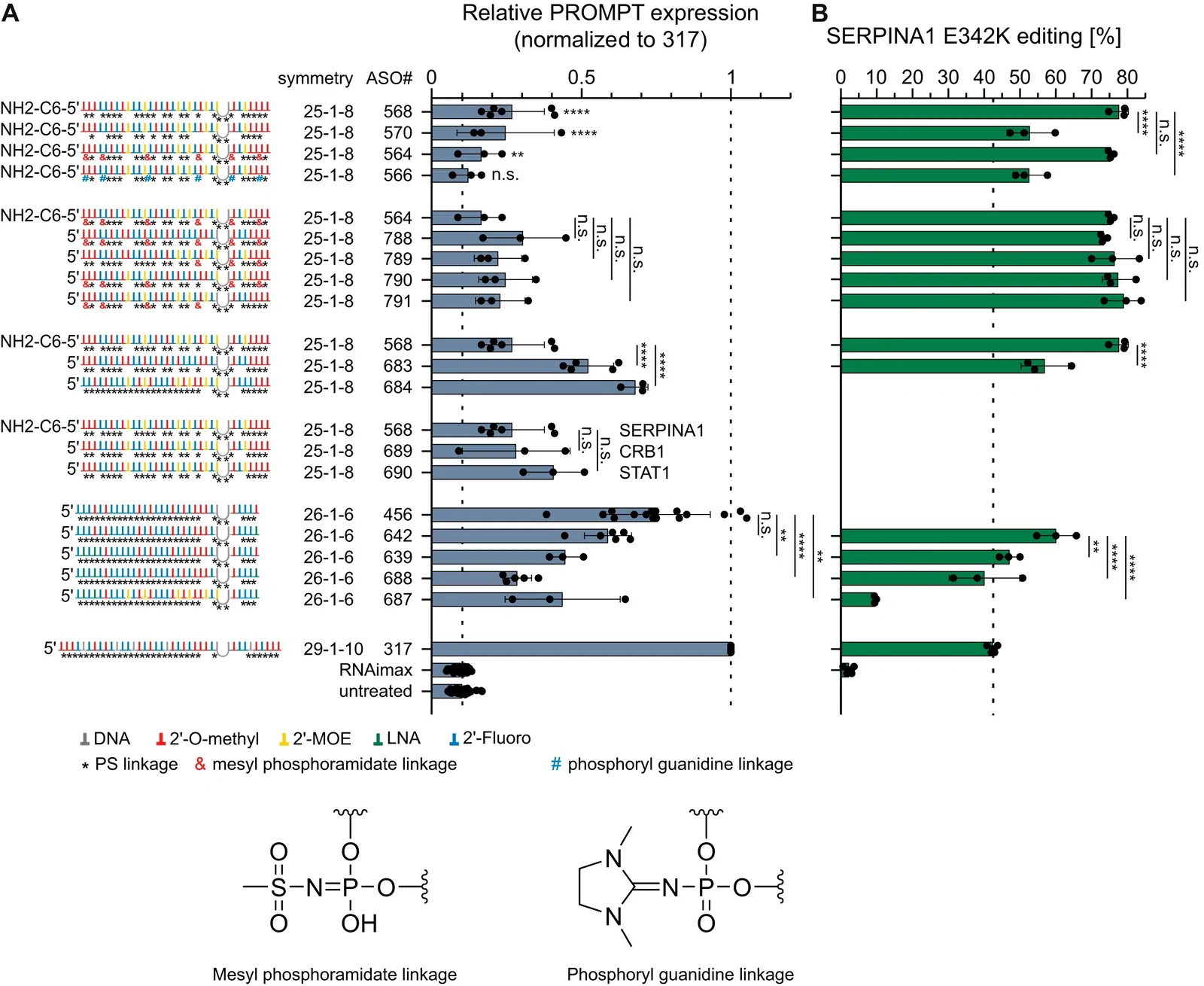
Combining several beneficial modifications abolishes RNA exosome interference and preserves editing efficiency. (**A**) Relative PROMPT expression after transfection with 50 nM of the respective oligonucleotide. Oligonucleotides differed in their 2′-modification patterns, their symmetries (corresponding to the counter base for RNA editing), their PS contents, and distributions. Further, a “good” pattern was transferred to two different targets and thus sequences. For all oligos, the symmetry is displayed as 5′-binding region-counter base-binding region-3′. (**B**) RNA editing yields to reverse the SERPINA1 E342K mutation 24 h after transfection with 25 nM of the respective oligonucleotide. Panels (A) and (B): asterisks mark significance (one-way ANOVA with a mixed effects model) as indicated by lines. Comparisons without lines refer to the untreated control. n.s. = not significant, *P < .05, **P < .01, ***P < .001, ^****^P < .0001. For comparisons with #317, a two-tailed *t*-test was performed.

## Discussion

In this study, we identified the RNA exosome as a previously underappreciated and modification-sensitive interaction partner of long, chemically stabilized RNA drugs. We extend the current understanding of RNA drug–protein interactions beyond well-studied pathways for RNase gapmers, splice switching oligonucleotides, and siRNAs. Our data show that single-stranded oligonucleotides exceeding 24 nt in length may bind to RNA exosome components, in particular the catalytic subunits DIS3 and EXOSC10, and may interfere with exosome activity in cells. This inhibitory interference correlates with stabilization of PROMPTs, perturbation of rRNA processing, and reduced cell viability, and is strongly modulated by the chemical modification pattern of the oligonucleotide.

Using proximity biotinylation, we observed preferential enrichment of DIS3 and EXOSC10 for oligonucleotides carrying 2′-OMe-modified 3′-wings compared to otherwise identical 2′-MOE-modified designs. This interaction correlated with a dose-dependent reduction in cell viability and with robust PROMPT accumulation, indicating that exosome binding is functionally relevant in cells. Importantly, neither degradation nor mislocalization of DIS3 or EXOSC10 was detected, distinguishing this mechanism from previously described gapmer-associated toxicities [13, 14]. The length requirement (≥24 nt) to induce increased PROMPT levels is highly consistent with structural data of the exosome [44, 46] and explains why this pathway escaped from recognition for approved RNA drugs. RNA substrates are threaded through the central channel toward the catalytic site of DIS3, spanning ~30–32 nt across DIS3 and the barrel structure [43]. Oligonucleotides shorter than 24 nt failed to induce PROMPT stabilization, whereas maximal effects were already reached at 30 nt. Together these data support a model in which nuclease-resistant oligonucleotides can function as non-degradable pseudo-substrates that sterically block RNA passage through the exosome rather than acting as inhibitors of isolated nucleases. Functionally, exosome inhibition manifested most prominently as stabilization of PROMPTs. Oligonucleotides with strong DIS3/EXOSC10 enrichment induced up to 10-fold increases in PROMPT levels in a dose-dependent manner, closely mirroring their toxicity profile and interactome data. In contrast, designs with 3′-terminal MOE modifications and reduced exosome binding showed only minor PROMPT stabilization. We also observed moderate accumulation of rRNA processing intermediates (ITS1 and 2) without major changes in mature rRNA levels. This phenotype is consistent with partial impairment of exosome-mediated rRNA maturation or degradation of the ITS1 fragment, yet less severe than phenotypes reported for structural exosome depletion [42] or certain toxic gapmers [12] and long splice switching oligonucleotides [40]. Nevertheless, PolyA-FISH experiments revealed substantial accumulation of polyadenylated RNA after treatment with long inhibitory oligonucleotides, reminiscent of exosome depletion phenotypes [48]. Interestingly, the increased signal was more pronounced in the cytosol than after EXOSC3 knockdown, potentially indicating secondary stress responses such as stress granule formation. Together, these findings suggest that long oligonucleotides can phenocopy partial exosome inhibition, leading to disturbed RNA surveillance and altered nuclear RNA homeostasis. Given the essential role of the exosome in RNA quality control and transcriptional regulation, even partial inhibition may contribute to cytotoxicity. However, we wish to mention that for a direct proof of the proposed mechanism of exosome inhibition, an *in vitro* oligonucleotide binding assay and an *in vitro* RNA turnover assay using the reconstituted 11-component complex would be required.

A central finding of this study is that exosome interference is highly sensitive to chemical modification patterns. Although 2′-OMe, 2′-F, DNA, LNA, and PS linkages are all widely used in approved or clinically advanced oligonucleotide therapeutics, their spatial arrangement within long oligonucleotides critically determines exosome interaction. Several principles emerge from our systematic analysis. First, the 3′-terminal chemistry is critical. A 3′-terminal 2′-OMe wing strongly promoted DIS3/EXOSC10 binding and PROMPT stabilization in the constructs tested here, whereas substitution with bulky 2′-MOE markedly reduced exosome inhibition. Notably, single MOE substitutions were insufficient; rather, multiple MOE residues acted synergistically, arguing against a single hotspot and in favor of cumulative steric or conformational effects. Second, the modification position matters beyond overall content. Shifting internal 2′-MOE, DNA, or LNA residues by few nucleotides substantially altered exosome inhibition, highlighting that the exosome-RNA interface is position-sensitive. Given the extensive backbone interactions observed in structural studies [43], small changes in local geometry or flexibility likely affect loading or channel passage. Third, the linkage chemistry contributes in a region-specific manner. High PS content, especially within the presumed channel-interacting 5′-half, enhanced PROMPT stabilization. This observation aligns with the known propensity of PS linkages to increase protein binding affinity [54]. Reduction of PS content synergized with beneficial ribose modifications, further attenuating exosome inhibition. Fourth, the sequence context modulates inhibition. Oligonucleotides sharing the same symmetry and modification pattern but targeting different transcripts exhibited up to six-fold differences in PROMPT accumulation. Collectively, these data indicate that exosome inhibition is not an intrinsic property of any single modification but rather an emergent feature of oligonucleotide length, oligonucleotide sequence, linkage chemistry, ribose chemistry, and their spatial arrangement.

ADAR-recruiting oligonucleotides are single-stranded, typically require lengths ≥30 nt and complex, mixed modification patterns for efficient editing. Our findings suggest a potential class-specific liability of this therapeutic modality: by virtue of their length and stabilization, these molecules fulfill the structural requirements for exosome engagement and inhibition. Importantly, we demonstrate that this liability is avoidable. By combining favorable 2′-modification patterns (notably incorporation of 2′-MOE), reduced or redistributed PS linkages, and in some cases sterically demanding alternative linkage chemistries such as mesyl phosphoramidate linkages, we identified highly efficient ADAR-recruiting oligonucleotides that induced only minimal PROMPT stabilization. These results show that high editing efficiency and low exosome inhibition are not mutually exclusive but can be achieved through chemical design. Given that protein binding is a central determinant of RNA drug pharmacology and toxicity, systematic profiling of protein interactomes should become part of the preclinical assessment of RNA drugs. PROMPT stabilization, in particular, may serve as a biomarker for exosome interference.

From a translational perspective, our findings are particularly relevant for emerging RNA base editing platforms entering clinical development. While first-in-human data for ADAR-recruiting oligonucleotides are promising [4], comprehensive characterization of class-specific protein interactions remains limited. Our study highlights that length-dependent engagement of essential RNA processing machineries can occur independently of target hybridization and sequence-specific effects. Rational placement of bulky ribose and linkage modifications provides a feasible path to mitigate this risk and widen the therapeutic window.

In summary, we identify exosome interference as a modification-, sequence-, and length-dependent off-target effect of long, single-stranded ASOs. By integrating interactome profiling, functional RNA analyses, and systematic chemical variation, we delineate structural principles that enable the design of ADAR-recruiting oligonucleotides with preserved efficacy and minimized interference with RNA surveillance. These insights contribute to the safer development of next-generation RNA base editing therapeutics.

## Supplementary Material

gkag990_Supplemental_Files

## Data Availability

The mass spectrometry proteomics data were deposited to the ProteomeXchange Consortium via the PRIDE partner repository with the dataset identifier PXD079777. Further information on additional data is available in the Supplementary information. Source data are provided with this paper. A table of all applied oligonucleotides disclosing their exact chemical modification pattern is found as Supplementary Data S1 file.
