# Supplementary material for: Chemically stabilized long antisense oligonucleotides perturb RNA exosome function
Source: Nucleic Acids Res. 2026 Oct 15;54(19):gkag990. doi: 10.1093/nar/gkag990 (PMC13669632; doi:10.1093/nar/gkag990)

Supplementary Information

Content

Supplementary Figures

Supplementary Tables

Appendix

qPCR Primer characterization

Uncropped images of western blots

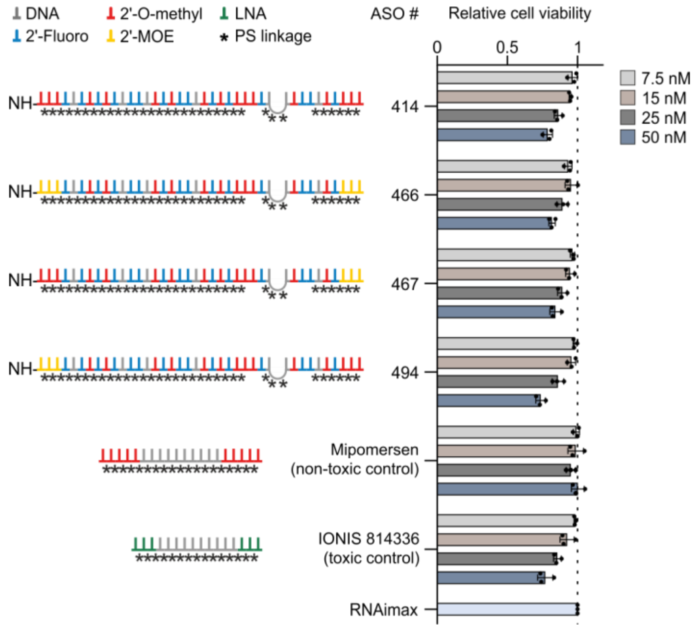

Fig. S1: Cell viability assay 24 h after oligonucleotide transfection. Shown are mean values, individual data points and the standard deviation. All signals were normalized to cells treated with RNAimax. As controls, two gapmer oligonucleotides (non-toxic and toxic) were included. In comparison to Fig. 1a, cell viability was higher 24 h after transfection indicating that toxic effects need more time to come into play. Further, the differences between the oligonucleotides are still smaller. N = 3.

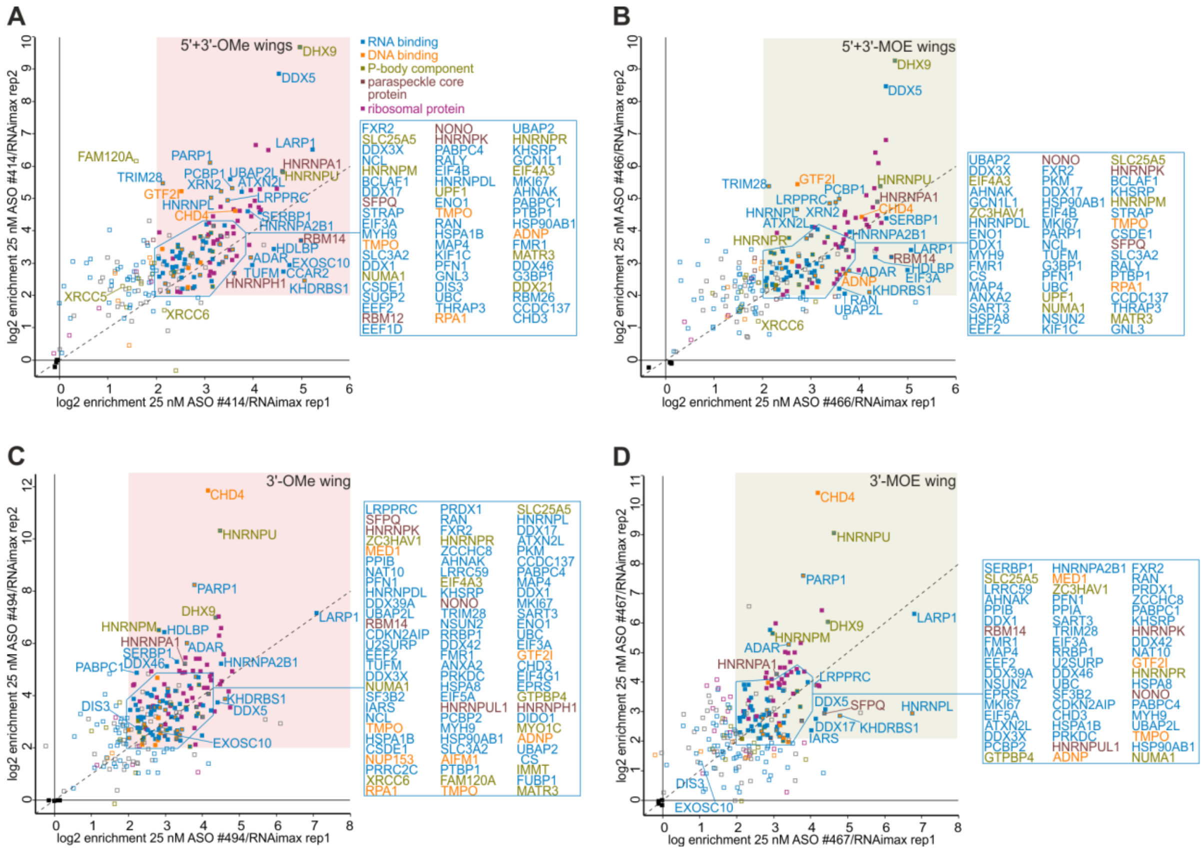

Fig. S2: Protein interactome data for the individual oligonucleotides against a negative control. As in Fig. 1b and c, 25 nM total oligonucleotide were transfected (15 nM BG-modified oligonucleotide and 10 nM NH-oligonucleotide) 24 h before fixation. In all panels, the relative log2 enrichment over a negative control (empty transfection) of two replicates with swapped SILAC labels is plotted. **a)** Protein interactions of oligonucleotide #414 containing 5'- and 3'-OMe wings. **b)** Protein interactions of oligonucleotide #466 containing 5'- and 3'-MOE wings. **c)** Protein interactions of oligonucleotide #494 containing a 5'-MOE and 3'-OMe wing. **d)** Protein interactions of oligonucleotide #467 containing a 5'-OMe and 3'-MOE wing. In all panels, all proteins were colored according to their GO terms (blue = RNA binding, orange = DNA binding, olive green = P-body component, dark red = paraspeckle core component, purple = ribosomal protein). All proteins with a log2 enrichment > 2 are highlighted by a colored background and mentioned with their name and additionally a few previously described oligonucleotide binders as well as DIS3 and EXOSC10 are displayed with their name.

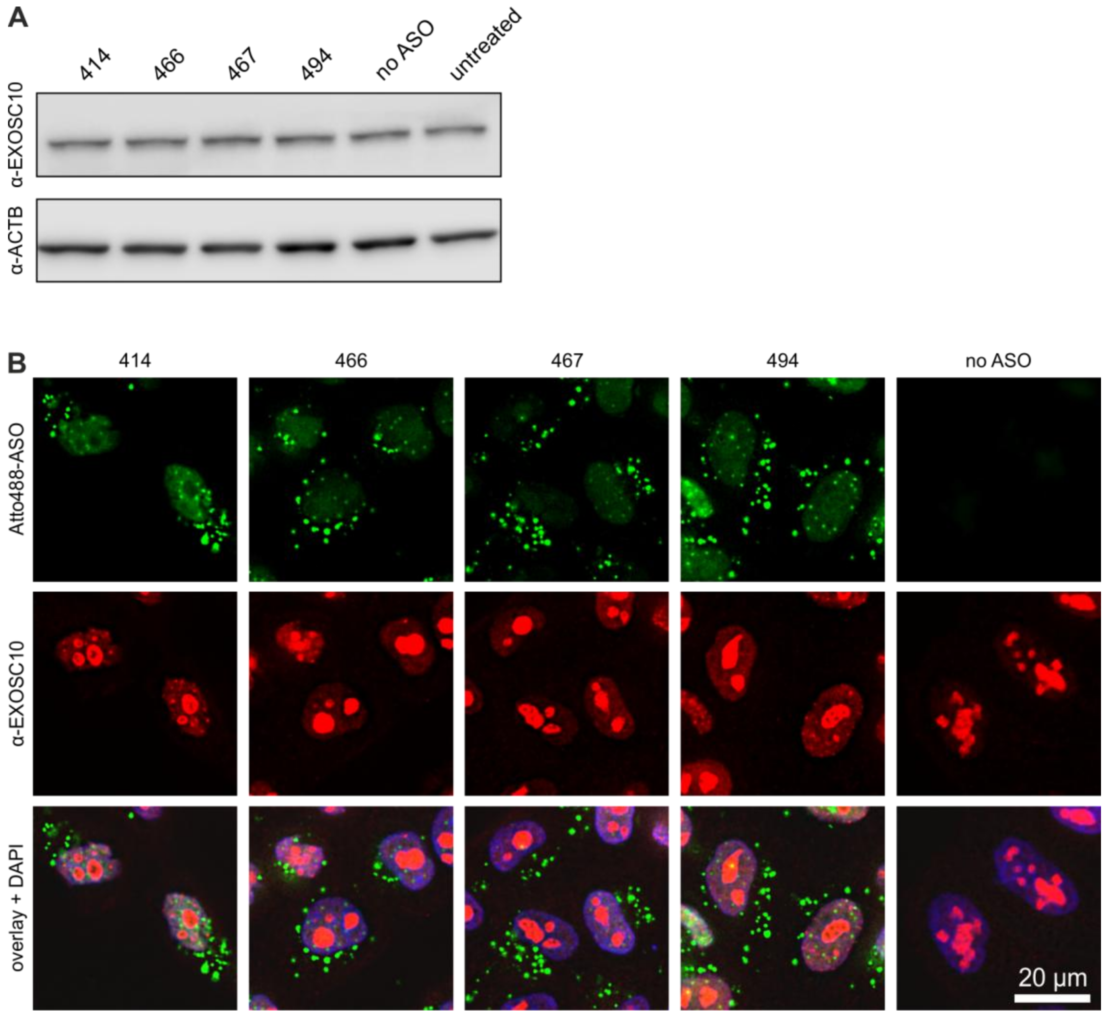

Fig. S3: Oligonucleotide transfection has no specific effect on EXOSC10 expression and localization. a) Western Blot against EXOSC10 and ACTB 24 h after transfection with 50 nM of the respective oligonucleotide. No protein degradation was observed for any of the transfected oligonucleotides. For the full plot and a second replicate, see in the appendix. **b)** Immunofluorescence microscopy against EXOSC10 24 h after transfection with 50 nM of the indicated oligonucleotide with 2 nM Atto-488 oligo spiked-in to investigate co-localization of EXOSC10 with the oligonucleotide. No clear changes in protein localization compared to an empty transfection (no oligonucleotide) were observed.

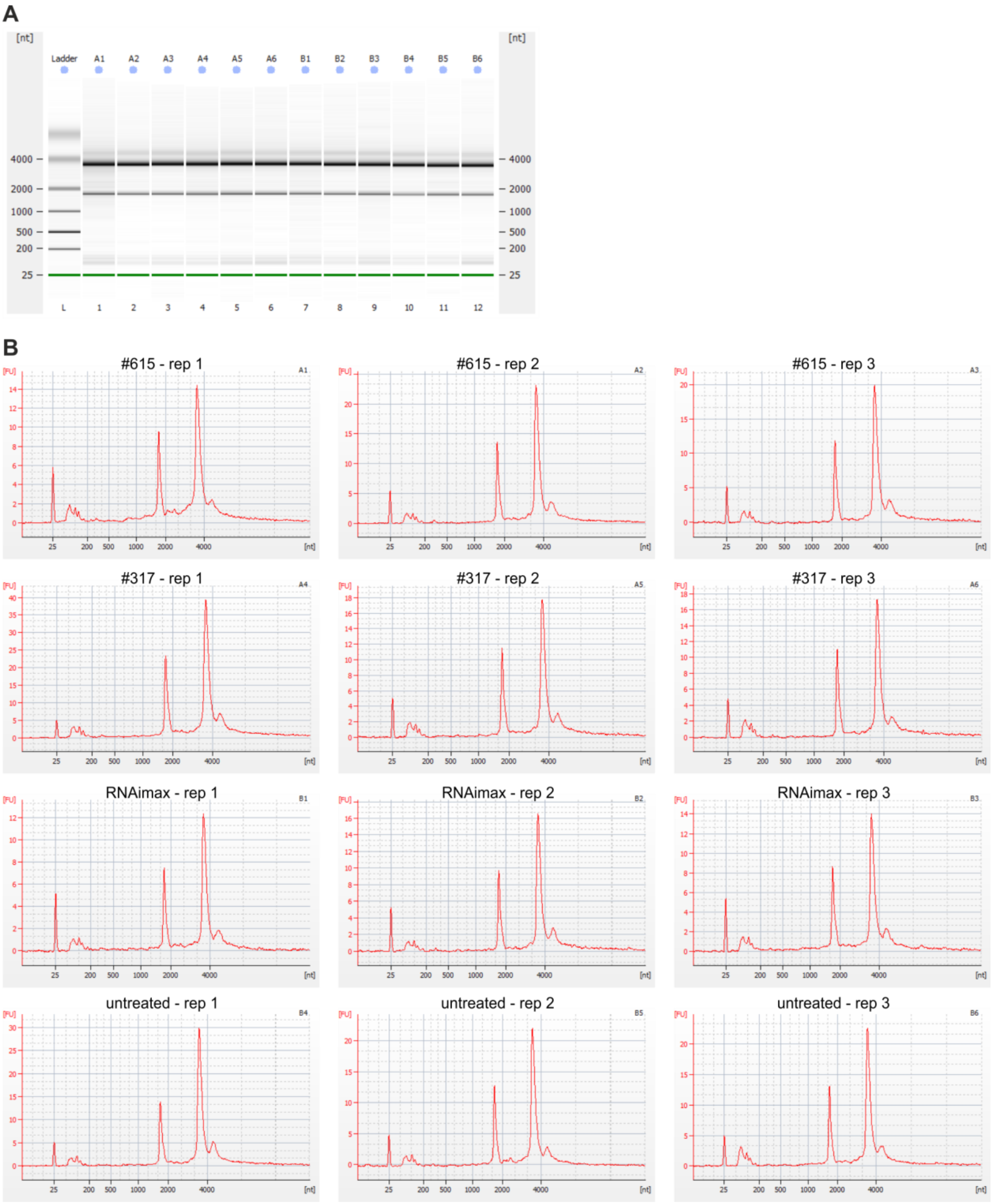

Fig. S4: Total RNA integrity after transfection of oligonucleotides inhibiting the RNA exosome. a) Bioanalyzer gel traces of all samples as labelled in b). **b)** Bioanalyzer chromatograms of all three replicates. The RIN values are plotted in Fig. 2b.

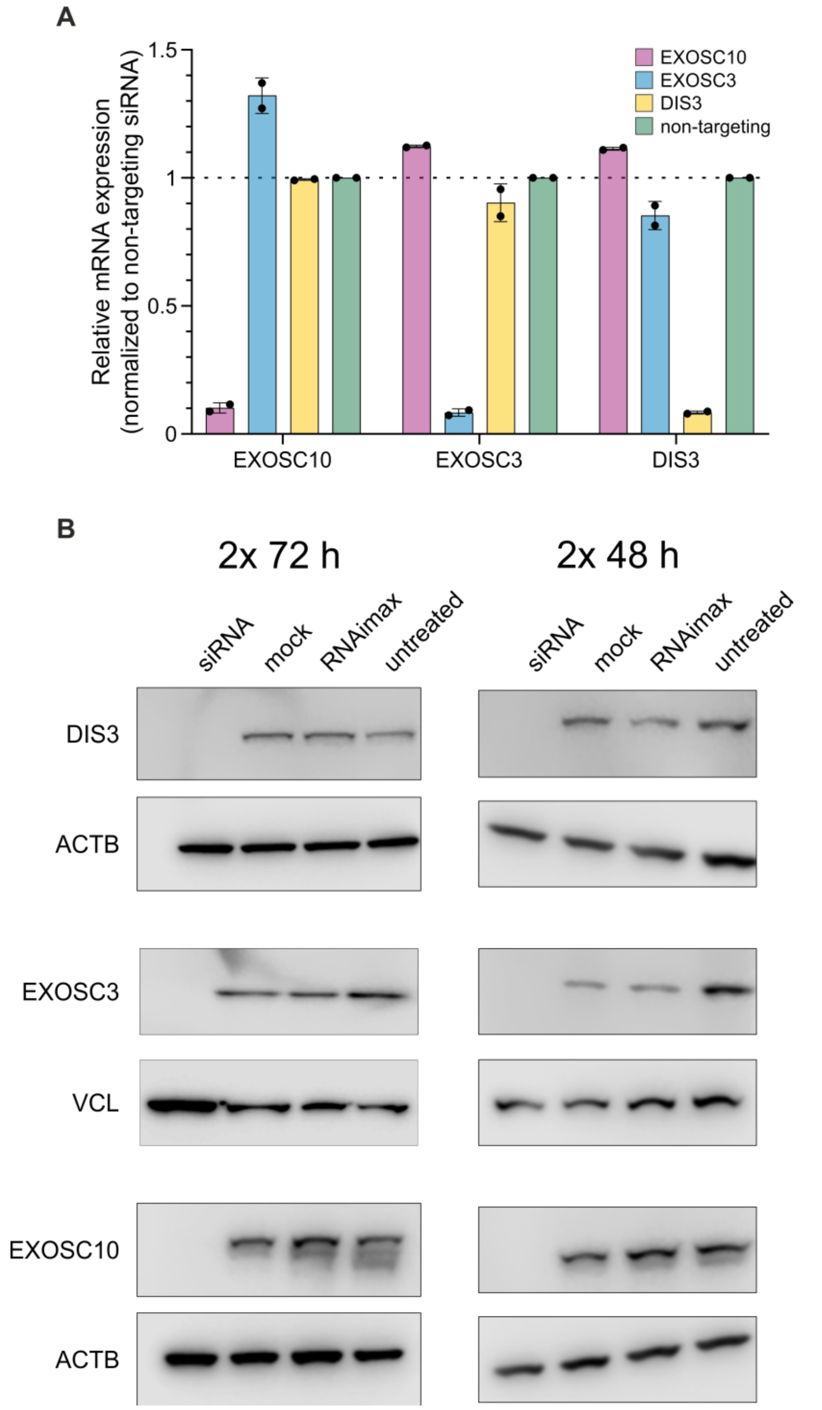

Fig. S5: RT-qPCR and Western Blots of DIS3, EXOSC3 and EXOSC10 after siRNA-mediated knockdown. 3 nM siRNA were reverse transfected for either 72 h or 48 h followed by another reverse transfection with the same concentration for the same time to achieve complete knockdown. **A)** RT-qPCR reveals siRNA specificity and efficiency. Around 90% knockdown efficiency were observed for all three siRNAs without affecting the other transcripts notably. **B)** In none of the cases, the targeted protein was still detectable by Western Blot. ACTB and VCL acted as loading controls. For the full images, see in the appendix.

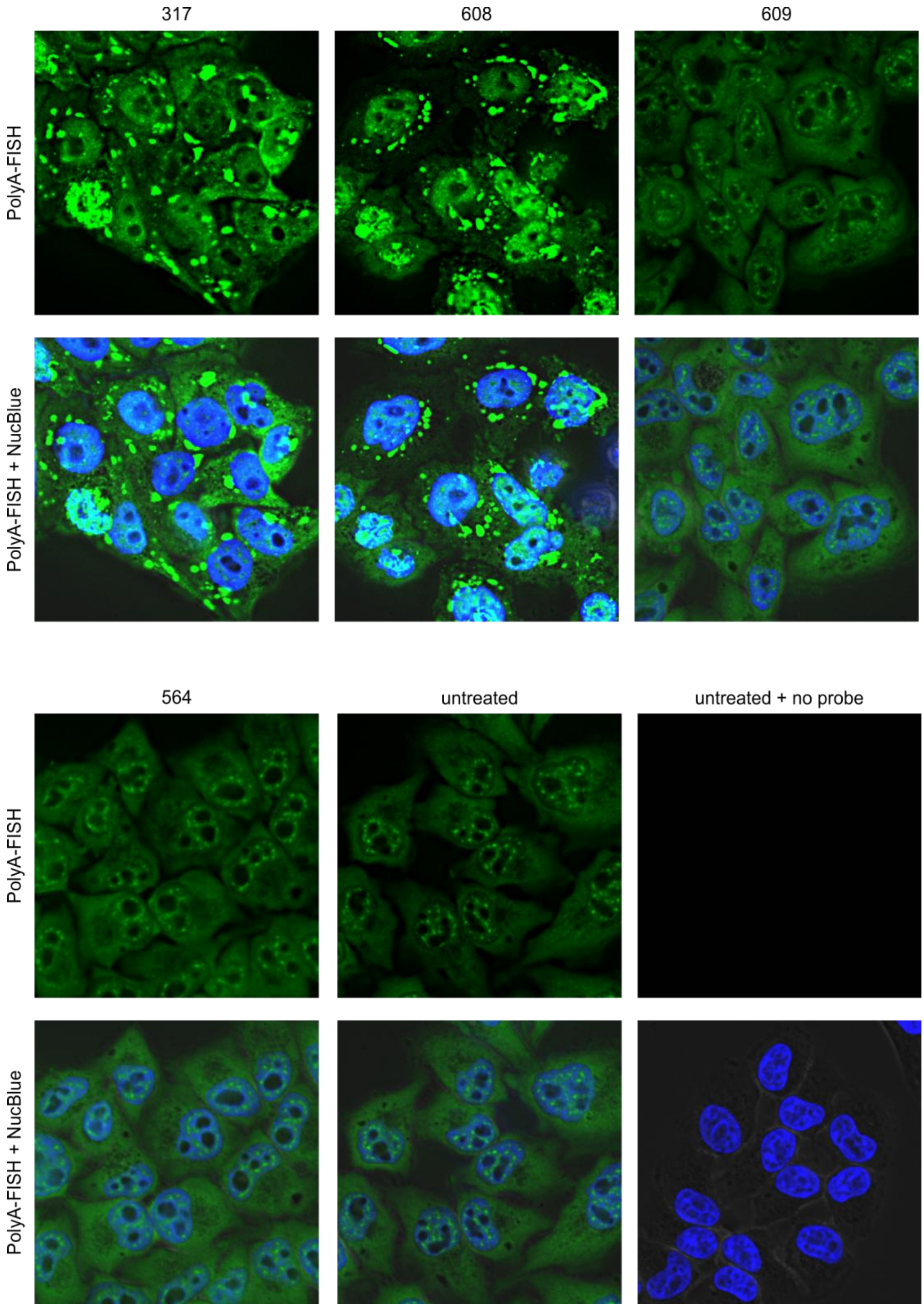

Fig. S6: PolyA FISH against total mRNA after oligonucleotide transfection reveals global dysregulation in mRNA homeostasis for toxic oligonucleotides. In all cases, 50 nM oligonucleotide were transfected for 24 h before fixation and total mRNA staining via FISH. Compared to the unperturbed condition, transfection of oligonucleotides #317 and #608 caused mRNA accumulation in cytoplasmic foci, potentially stress granules. In contrast, oligonucleotides #609 and #564 did not cause this effect.

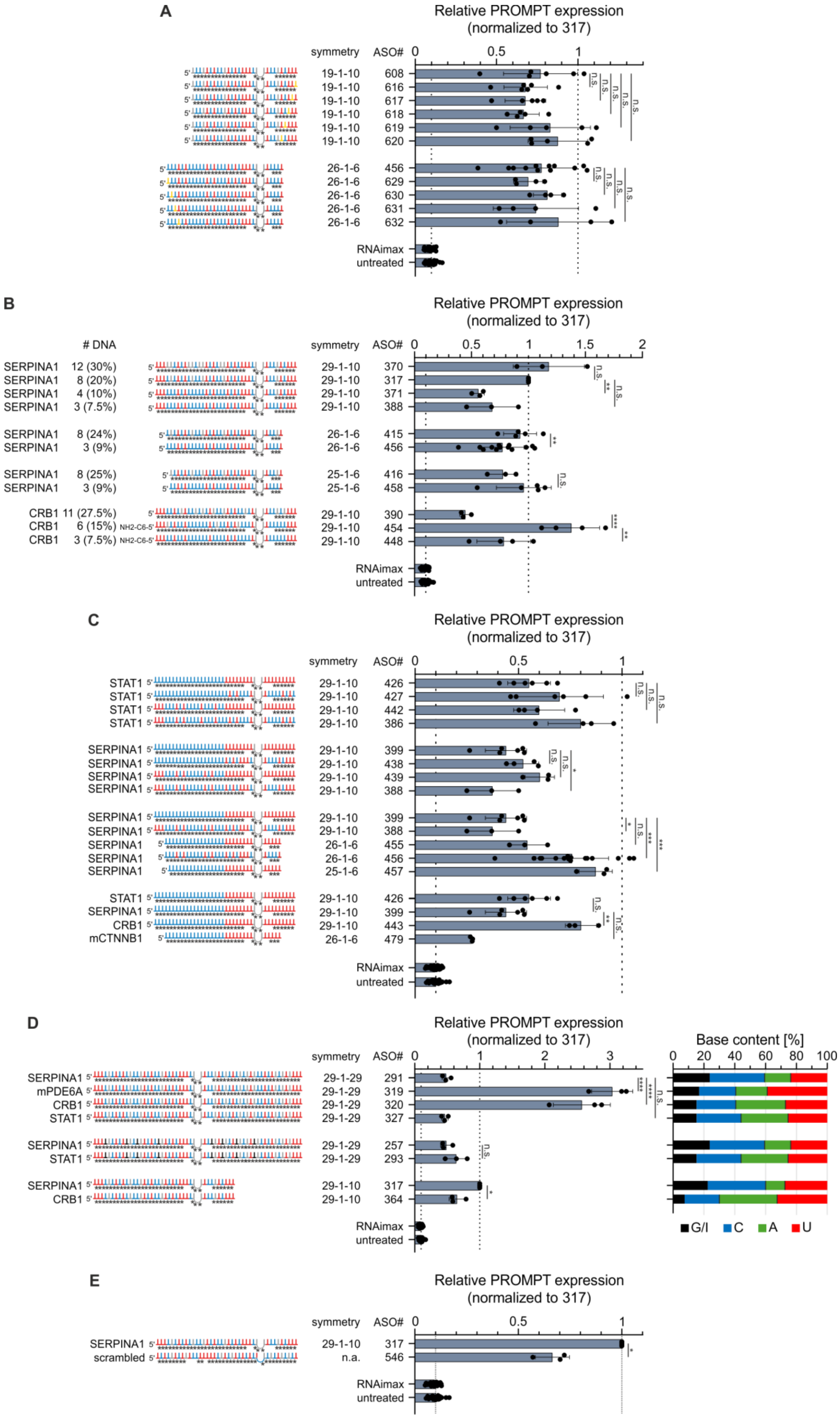
A
Relative PROMPT expression (normalized to 317)
symmetry ASO#
0 0.5 1
19-1-10 608
19-1-10 616
19-1-10 617
19-1-10 618
19-1-10 619
19-1-10 620
26-1-6 456
26-1-6 629
26-1-6 630
26-1-6 631
26-1-6 632
RNAimax
untreated
n.s.
B
Relative PROMPT expression (normalized to 317)
DNA
symmetry ASO#
0 0.5 1 1.5 2
SERPINA1 12 (30%) 29-1-10 370
SERPINA1 8 (20%) 29-1-10 317
SERPINA1 4 (10%) 29-1-10 371
SERPINA1 3 (7.5%) 29-1-10 388
SERPINA1 8 (24%) 26-1-6 415
SERPINA1 3 (9%) 26-1-6 456
SERPINA1 8 (25%) 25-1-6 416
SERPINA1 3 (9%) 25-1-6 458
CRB1 11 (27.5%) 29-1-10 390
CRB1 6 (15%) NH2-C6-5' 29-1-10 454
CRB1 3 (7.5%) NH2-C6-5' 29-1-10 448
RNAimax
untreated
C
Relative PROMPT expression (normalized to 317)
symmetry ASO#
0 0.5 1
STAT1 29-1-10 426
STAT1 29-1-10 427
STAT1 29-1-10 442
STAT1 29-1-10 386
SERPINA1 29-1-10 399
SERPINA1 29-1-10 438
SERPINA1 29-1-10 439
SERPINA1 29-1-10 388
SERPINA1 29-1-10 399
SERPINA1 29-1-10 388
SERPINA1 26-1-6 455
SERPINA1 26-1-6 456
SERPINA1 25-1-6 457
STAT1 29-1-10 426
SERPINA1 29-1-10 399
CRB1 29-1-10 443
mCTNNB1 26-1-6 479
RNAimax
untreated
D
Relative PROMPT expression (normalized to 317)
symmetry ASO#
0 1 2 3
SERPINA1 29-1-29 291
mPDE6A 29-1-29 319
CRB1 29-1-29 320
STAT1 29-1-29 327
SERPINA1 29-1-29 257
STAT1 29-1-29 293
SERPINA1 29-1-10 317
CRB1 29-1-10 364
RNAimax
untreated
Base content [%]
0 20 40 60 80 100
G/I C A U
E
Relative PROMPT expression (normalized to 317)
symmetry ASO#
0 0.5 1
SERPINA1 29-1-10 317
scrambled n.a. 546
RNAimax
untreated

Fig. S7: Relative PROMPT expression after 50 nM oligonucleotide transfection for 24 h. Expression was internally normalized to *GAPDH* and *ACTB* expression levels and afterwards referenced to oligonucleotide #317. In all panels, the mean is shown with individual data points and the standard deviation. **a)** Oligonucleotides for different targets were tested with the same design patterns as well as mixed versions of the patterns to investigate the influence of specific oligonucleotide regions on exosome inhibition. **b)** Oligonucleotides with different sequences but the same modification pattern were tested. In the right panel, the content of all four bases is shown. **c)** Oligonucleotides with differences in the DNA content were tested for exosome inhibition. **d)** MOE-walk through the 3'-wing of a 30mer oligonucleotide and through the 5'-wing of a 33mer oligonucleotide was performed. No hotspot site could be identified that reduces RNA exosome inhibition notably. **e)** Comparison of PROMPT expression between the reference oligonucleotide and a scrambled version that contains the same amount of each base, PS-modifications and 2'-modifications than oligonucleotide #317. Asterisks mark significance (one-way ANOVA with a mixed effects model) as indicated by lines. n.s. = not significant, * $p < 0.05$, ** $p < 0.01$, *** $p < 0.001$, **** $p < 0.0001$. For comparisons with #317, a two-tailed t-test was performed.

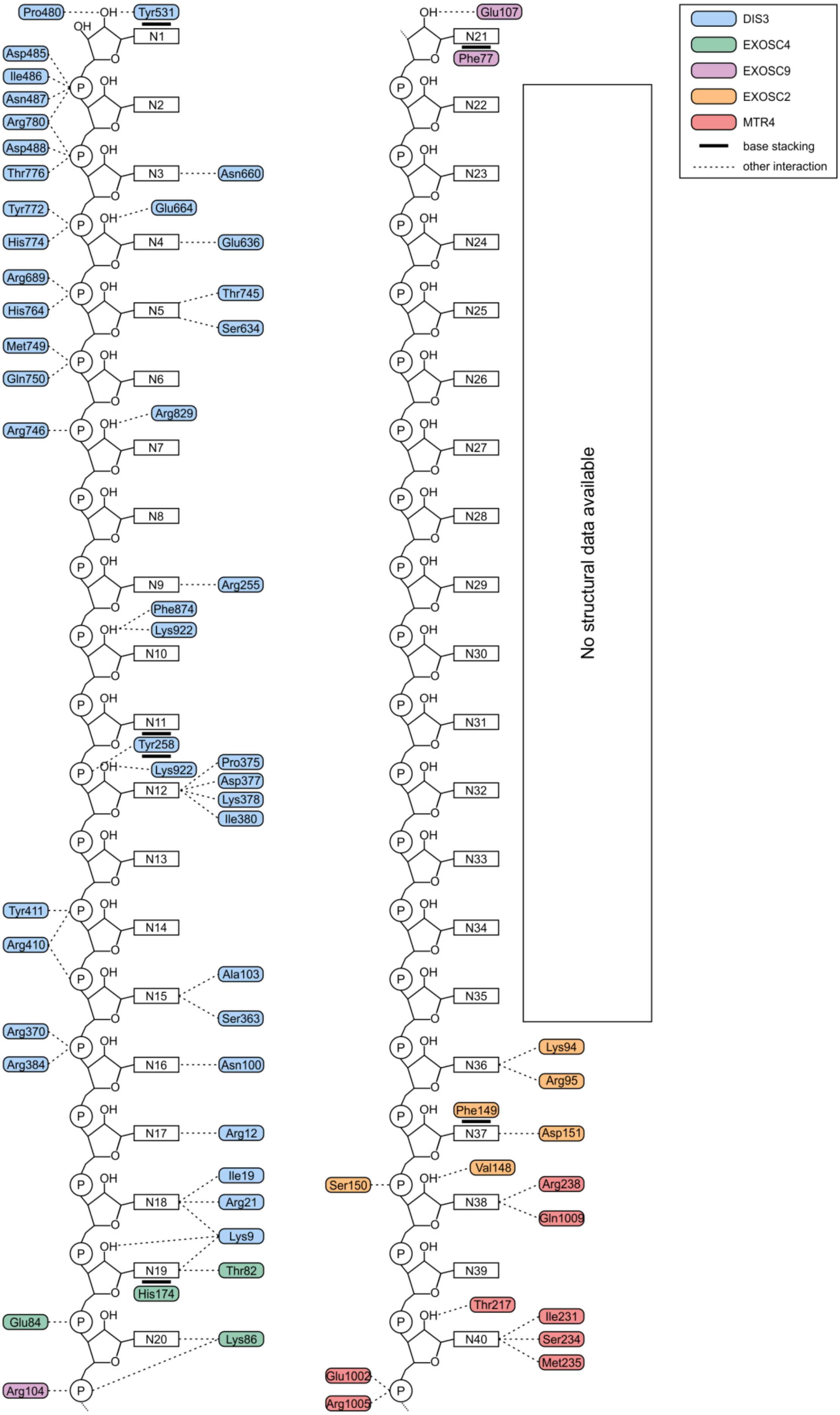

DIS3
EXOSC4
EXOSC9
EXOSC2
MTR4
base stacking
other interaction
Pro480
OH
Tyr531
N1
Asp485
Ile486
Asn487
Arg780
N2
Asp488
Thr776
N3
Asn660
Tyr772
Glu664
His774
N4
Glu636
Arg689
His764
N5
Thr745
Ser634
Met749
Gln750
N6
Arg746
Arg829
N7
N8
N9
Arg255
Phe874
Lys922
N10
N11
Tyr258
Lys922
Pro375
Asp377
Lys378
Ile380
N12
N13
Tyr411
Arg410
N14
N15
Ala103
Ser363
Arg370
Arg384
N16
Asn100
N17
Arg12
Ile19
N18
Arg21
Lys9
N19
Thr82
His174
Glu84
N20
Lys86
Arg104
P
Glu107
N21
Phe77
N22
N23
N24
N25
N26
N27
N28
N29
N30
N31
N32
N33
N34
N35
No structural data available
N36
Lys94
Arg95
Phe149
N37
Asp151
Val148
Ser150
N38
Arg238
Gln1009
N39
Thr217
N40
Ile231
Ser234
Met235
Glu1002
Arg1005

Fig. S8: Interaction map between the RNA exosome complex and an RNA substrate. The crystal structure was obtained from (Weick et al. (45), PDB accession code 6D6R) and analyzed as follows. Potential interactions were filtered based on proximity between the nucleic acid substrate and the protein within 3.5 Å and subsequently classified as base stacking or other interactions with either the base, the 2'-OH of the ribose or the phosphate backbone. Only direct contacts between amino acids and the nucleic acid were considered, no water- or metal-mediated contacts. Blue = DIS3, green = EXOSC4, purple = EXOSC9, orange = EXOSC2, red = MTR4. MTR4 can act as a proxy for EXOSC10 position since both proteins compete for the position on top of the barrel where the RNA substrate enters. The white box indicates a region without any information on the RNA in the crystal structure and thus no contact sites could be determined.

Supplementary Tables:

Supplementary Table 1: Antibodies

Antibody (antigen size)	Supplier, order number	Origin	Dilution western blot	Dilution Immunofluorescence
ACTB (42 kDa)	SigmaAldrich, A5441	Mouse	1:5,000	-
DIS3 (100 kDa)	Santa Cruz Biotech., sc-398663	Mouse	1:500	1:250
EXOSC3 (31 kDa)	Santa Cruz Biotech., sc-166568	Mouse	1:500	-
EXOSC10 (110 kDa)	Santa Cruz Biotech., sc-374595	Mouse	1:500	1:250
VCL (124 kDa)	Cell Signalling, 13901	Rabbit	1:1,000	-
α-Mouse Alexa Fluor 594	Cell Signalling, 8890S	Goat	-	1:1,000
α-Mouse HRP	Jackson Immuno Research Laboratories, 115-035-003	Goat	1:10,000	-
α-Rabbit HRP	Jackson Immuno Research Laboratories, 111-035-003	Goat	1:10,000	-

Supplementary Table 2: SILAC experimentsd

Exp. No.	Description		Figures
R7	240 nM SNAP-BASU-His	L: RNAimax<br>M: 15 nM BG-modified oligo #414 + 10 nM oligo #414<br>H: 15 nM BG-modified oligo #466 + 10 nM oligo #466	1B, S2A, S2B
R8	240 nM SNAP-BASU-His	L: 15 nM BG-modified oligo #466 + 10 nM oligo #466<br>M: RNAimax<br>H: 15 nM BG-modified oligo #414 + 10 nM oligo #414	1B, S2A, S2B
R9	240 nM SNAP-BASU-His	L: RNAimax<br>M: 15 nM BG-modified oligo #467 + 10 nM oligo #467<br>H: 15 nM BG-modified oligo #494 + 10 nM oligo #494	1C, S2C, S2D
R10	240 nM SNAP-BASU-His	L: 15 nM BG-modified oligo #494 + 10 nM oligo #494<br>M: RNAimax<br>H: 15 nM BG-modified oligo #467 + 10 nM oligo #467	1C, S2C, S2D

Supplementary Table 3: qPCR primers

Target RNA	Amplicon Size	Forward Primer Sequence	Reverse Primer Sequence
5.8S rRNA	103	CTTAGCGGTGGATCACTCGG	TGCGTTCGAAGTGTCGATGAT
ITS2	113	GTGGAAGGTCCCGTGCC	ACCCTTCCCCTTCCGGAC
18S rRNA	94	CCTGGTTGATCCTGCCAGTAG	GCCATTCGCAGTTTCACTGT
ITS1	91	CGTGGTGTGAAACCTTCCGA	CCCAAGAGGAGAGGGGGTT
ACTB	91	CGGGACCTGACTGACTAC	TAATGTCACGCACGATTTCC
DIS3	103	ACTGCACCAGATTGATGTTCTTG	TTTATATACGGGGGCACTGCGA
EXOSC10	119	GTCTCTCAGGCAGCGAAGTT	GCTGCCTTCTTCTTGACAGC
EXOSC3	114	ACCAAATGTGCAGGTTGGAGA	ACACCCATTCCATTGGCTCG
GAPDH	96	CAACAGCCTCAAGATCATCAG	CCTTCCACGATACCAAAGTTG
PPM1G PROMPT	120	TCCCACCCAGAATAGGCTGT	GCCATGCGAGGCCTATATGA
SEPHS1 PROMPT	118	ACTTCCTACCACGCACATGA	GACTCCCAGCATCTGTCCTTC
STK11IP PROMPT	105	CACGAGTTTGGAGGGAGTCT	TATCCGCCGTAGGATGGACT

Supplementary Table 4: RNA Editing analysis primers

Primer name	Sequence
SERPINA1_fwd	ACCCACGATATCATCACCAA
SERPINA1_rev	CTTTTCCCATGAAGAGGGGAG
SERPINA1_sense	CCGTGCATAAGGCTGTGCTGACCATCGACCAGAAAGGGACTGAAGCTGCTGGGGCCATGTTTTTCCC

Appendix:

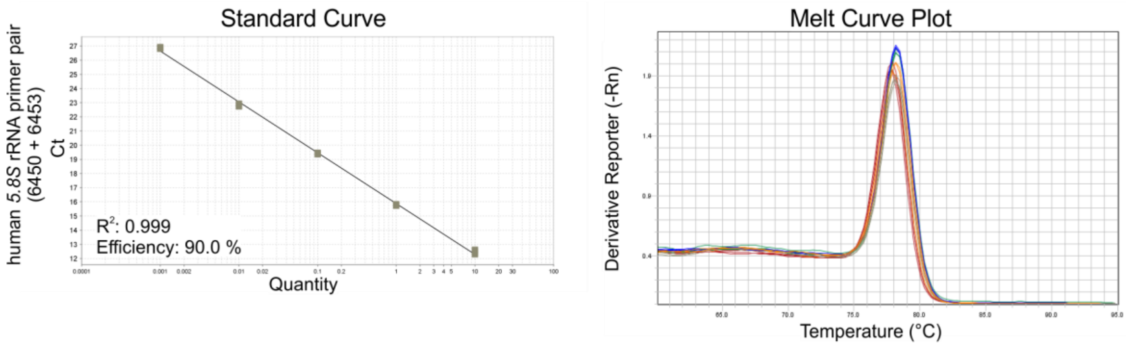

human 5.8S rRNA primer pair
(6450 + 6453)
Standard Curve
Ct
R²: 0.999
Efficiency: 90.0 %
Quantity
Melt Curve Plot
Derivative Reporter (-Rn)
Temperature (°C)

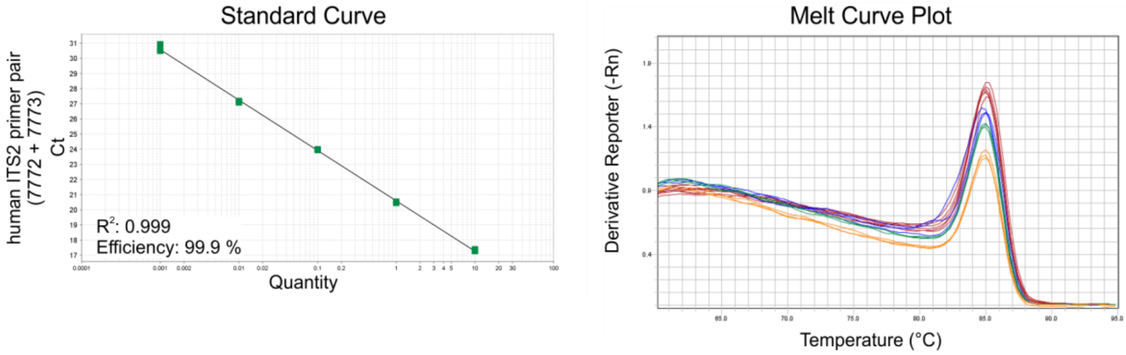

human ITS2 primer pair
(7772 + 7773)
Standard Curve
Ct
R²: 0.999
Efficiency: 99.9 %
Quantity
Melt Curve Plot
Derivative Reporter (-Rn)
Temperature (°C)

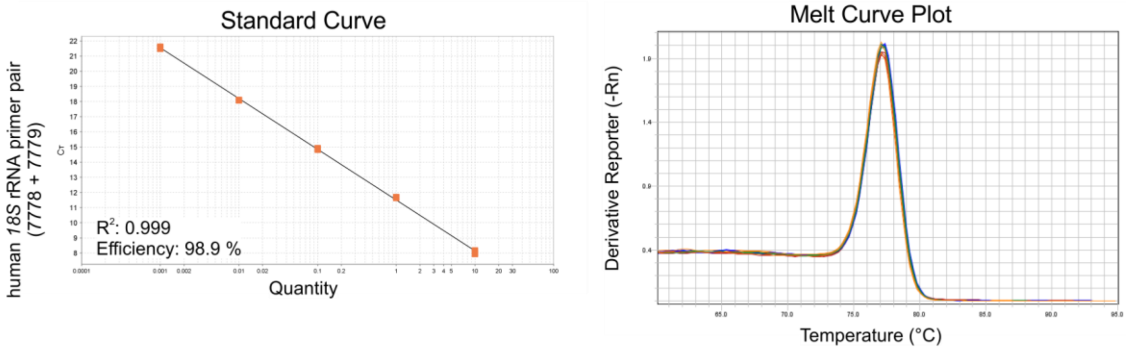

human 18S rRNA primer pair
(7778 + 7779)
Standard Curve
R²: 0.999
Efficiency: 98.9 %
Quantity
Melt Curve Plot
Derivative Reporter (-Rn)
Temperature (°C)

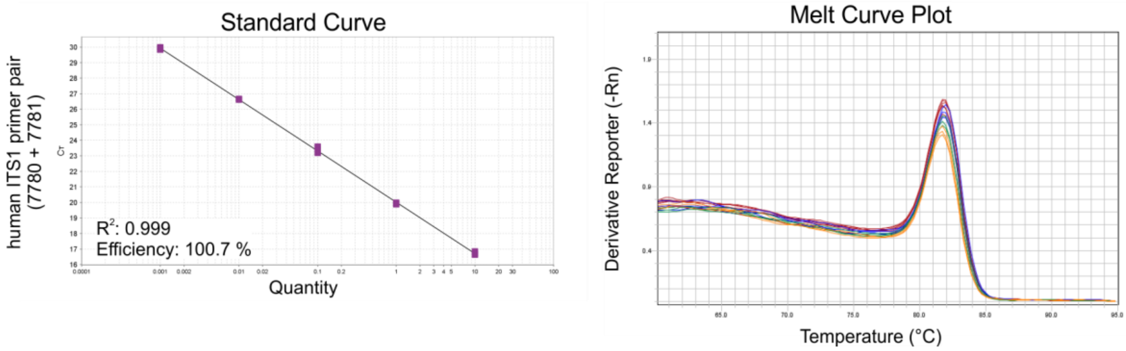

human ITS1 primer pair
(7780 + 7781)
Standard Curve
R²: 0.999
Efficiency: 100.7 %
Quantity
Melt Curve Plot
Derivative Reporter (-Rn)
Temperature (°C)

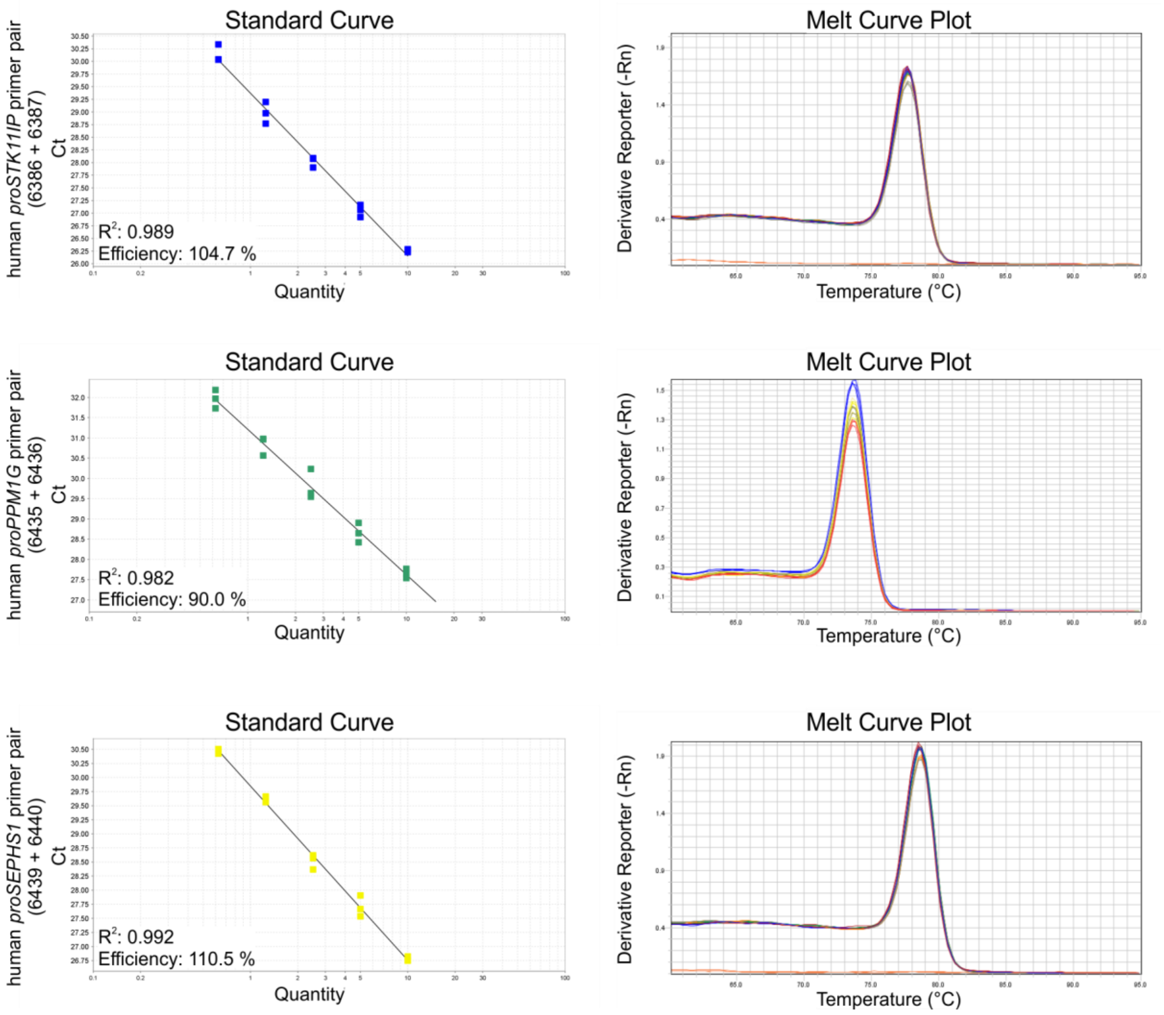

Standard Curve
human proSTK11IP primer pair
(6386 + 6387)
Ct
R²: 0.989
Efficiency: 104.7 %
Quantity
Melt Curve Plot
Derivative Reporter (-Rn)
Temperature (°C)
Standard Curve
human proPPM1G primer pair
(6435 + 6436)
Ct
R²: 0.982
Efficiency: 90.0 %
Quantity
Melt Curve Plot
Derivative Reporter (-Rn)
Temperature (°C)
Standard Curve
human proSEPHS1 primer pair
(6439 + 6440)
Ct
R²: 0.992
Efficiency: 110.5 %
Quantity
Melt Curve Plot
Derivative Reporter (-Rn)
Temperature (°C)

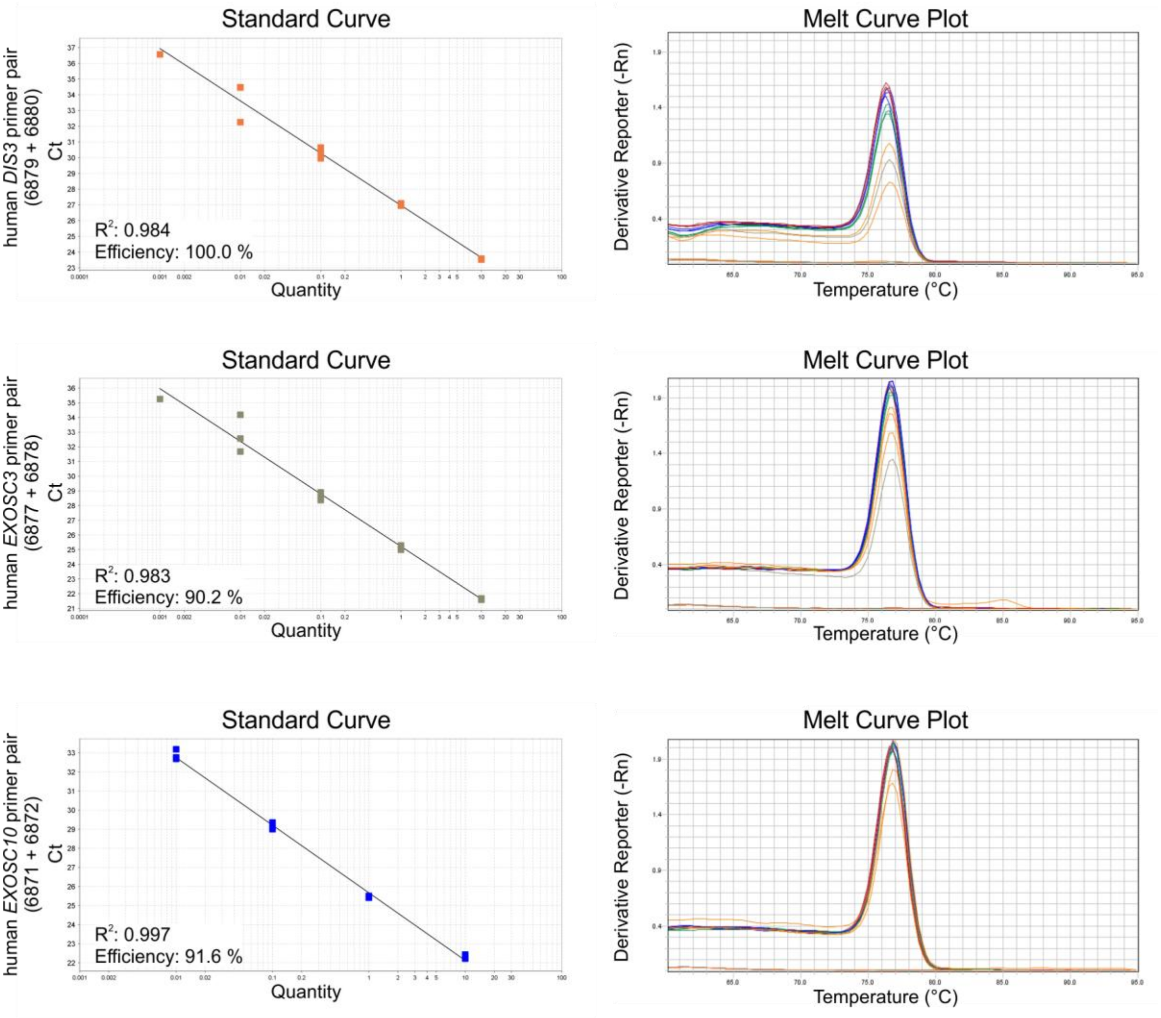

Standard Curve
Melt Curve Plot
human DIS3 primer pair (6879 + 6880)
Ct
R²: 0.984
Efficiency: 100.0 %
Quantity
Derivative Reporter (-Rn)
Temperature (°C)
Standard Curve
Melt Curve Plot
human EXOSC3 primer pair (6877 + 6878)
Ct
R²: 0.983
Efficiency: 90.2 %
Quantity
Derivative Reporter (-Rn)
Temperature (°C)
Standard Curve
Melt Curve Plot
human EXOSC10 primer pair (6871 + 6872)
Ct
R²: 0.997
Efficiency: 91.6 %
Quantity
Derivative Reporter (-Rn)
Temperature (°C)

Fig. 1 DIS3

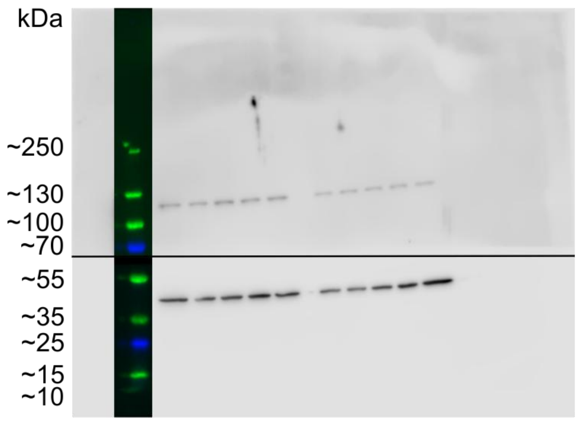

rep2 for Fig. 1 DIS3

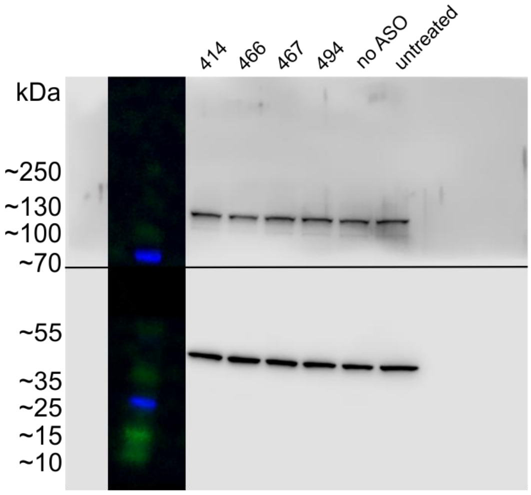

Fig. S3 EXOSC10 rep1

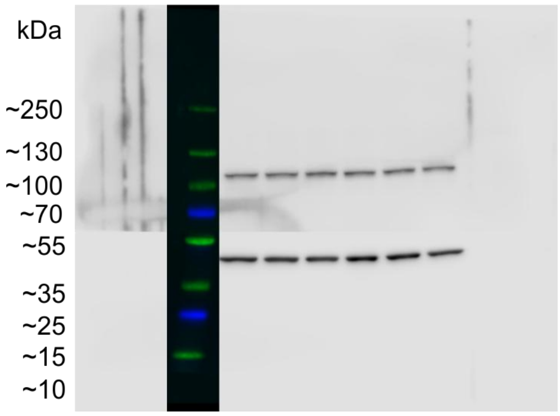

Fig. S3 EXOSC10 rep2

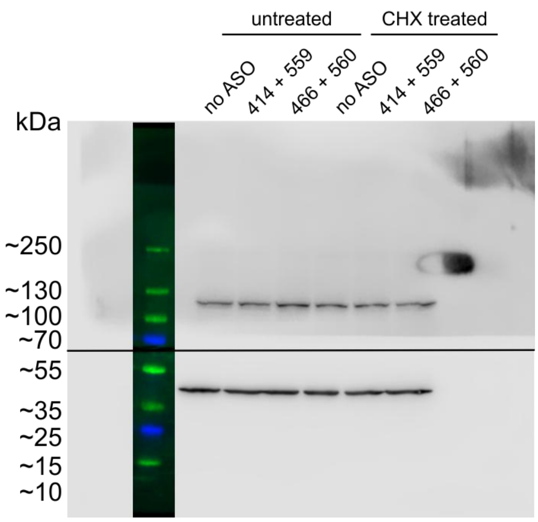

Fig. S5 DIS3 2x 48 h

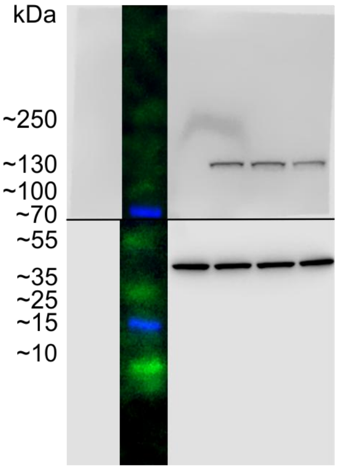

Fig. S5 DIS3 2x 72 h

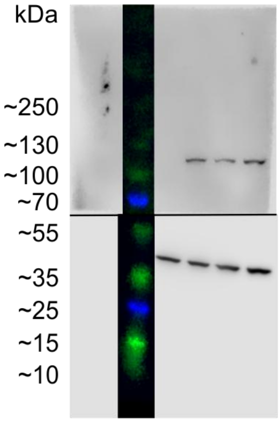

Fig. S5 EXOSC3 2x 48 h

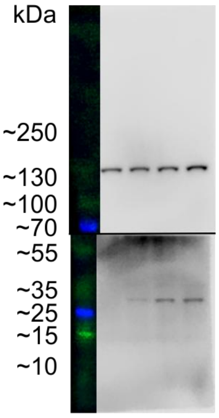

Fig. S5 EXOSC3 2x 72 h

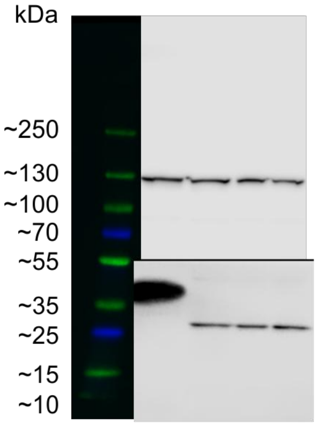

Fig. S5 EXOSC10 2x 48 h

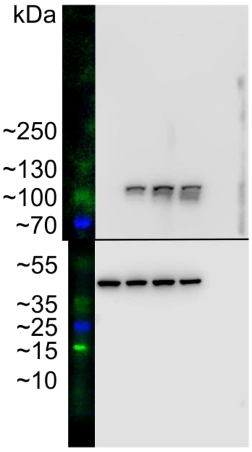

Fig. S5 EXOSC10 2x 72 h

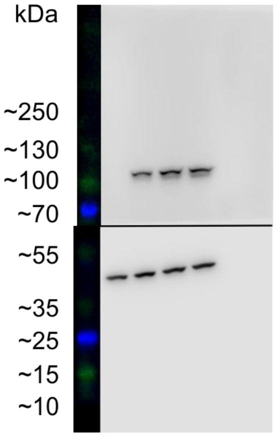

Supplement: Supplementary file 1 [file gkag990_supplemental_files.zip › Supplementary_Information_revised_final.pdf]
